# Autogenous platelet concentrates for treatment of intrabony defects—A systematic review with meta‐analysis

**DOI:** 10.1111/prd.12598

**Published:** 2024-10-19

**Authors:** Richard J. Miron, Vittorio Moraschini, Nathan Estrin, Jamil Awad Shibli, Raluca Cosgarea, Karin Jepsen, Pia‐Merete Jervøe‐Storm, Hom‐Lay Wang, Anton Sculean, Søren Jepsen

**Affiliations:** ^1^ Department of Periodontology University of Bern Bern Switzerland; ^2^ Department of Oral Surgery, School of Dentistry, Fluminense Federal University Niterói Rio de Janeiro Brazil; ^3^ School of Dental Medicine Lake Erie College of Osteopathic Medicine Bradenton Florida USA; ^4^ Department of Implant Dentistry, School of Dentistry Guarulhos University Guarulhos Brazil; ^5^ Department of Periodontology, Operative and Preventive Dentistry University of Bonn Bonn Germany; ^6^ Department of Periodontology University of Marburg Germany; ^7^ Faculty of Dentistry University Iuliu Hatieganu Cluj‐Napoca Romania; ^8^ Department of Periodontics and Oral Medicine University of Michigan Ann Arbor Michigan USA

**Keywords:** advanced‐PRF, horizontal centrifugation, intrabony defect, leukocyte and platelet‐rich fibrin, L‐PRF, meta‐analysis, periodontal regeneration, periodontitis, systematic review

## Abstract

To provide an overview of the use of autogenous platelet concentrates (APCs) in periodontal regeneration and to conduct a systematic review (SR) of the treatment outcomes of periodontal intrabony defects by using platelet‐rich fibrin (PRF) compared with other commonly utilized modalities. The eligibility criteria comprised randomized controlled trials (RCTs) comparing the clinical outcomes of PRF with that of other modalities. Studies were classified into 21 categories and into five different groups as follows: Group I (1) open flap debridement (OFD) alone versus OFD/PRF, (2) OFD versus Titanium‐PRF (T‐PRF) Group II, (3) Comparative PRF protocols (PRF vs. T‐PRF), Group III (Comparative Studies to PRF): (4) OFD/PRP versus OFD/PRF, (5) OFD/bone graft(BG)/PRGF versus OFD/BG/PRF, (6) OFD/EMD versus OFD/PRF, (7) OFD/BG/EMD versus OFD/BG/PRF, (8) OFD/collagen membrane (CM) versus OFD/PRF, (9) OFD/BG/BM versus OFD/BG/PRF, (10) OFD/BG versus OFD/PRF, Group IV (Addition of PRF to treatment groups) (11) OFD/BG versus OFD/BG/PRF, (12) OFD/GTR versus OFD/GTR + PRF (13) OFD/EMD versus OFD/EMD/PRF (14) OFD/BG/BM versus OFD/BG/BM/PRF, Group V (Addition of Biomaterial/Biomolecule to PRF): OFD/PRF versus … (15) OFD/PRF/BG, (16) OFD/PRF/antibiotic, (17) OFD/PRF/Metformin, (18) OFD/PRF/Bisphosphonates, (19) OFD/PRF/Statins, (20) OFD/BG/PRF versus OFD/BG/PRF/Statins, and (21) OFD/PRF/low‐level laser therapy (LLLT). Weighted means and forest plots were calculated for probing pocket depth (PPD), clinical attachment level (CAL), and radiographic bone fill (RBF). From 596 records identified, 55 RCTs were included. Group I: The use of OFD/PRF statistically significantly reduced PPD and improved CAL and RBF when compared to OFD. Group II: A significant difference between various PRF protocols was only observed for PPD. Group III: No significant advantage was found when comparing OFD/PRF to the following groups: OFD/PRP, OFD/EMD, OFD/BM, or OFD/BG. Group IV: The addition of PRF to OFD/BG led to significant improvements in PPD, CAL and RBF compared with OFD/BG alone. Group V: The addition of either a BG as well as three of the following biomolecules (metformin, bisphosphonates, and statins) to OFD/PRF led to statistically significant improvements in PPD, CAL, and/or RBF when compared to OFD/PRF alone. The use of PRF significantly improved clinical outcomes in intrabony defects when compared to OFD alone. Similar results were observed when OFD/PRF was compared with OFD/BG, OFD/EMD, OFD/PRP, and OFD/BM. The addition of PRF to a bone grafting material as well as the addition of various small biomolecules to PRF may offer additional clinical advantages, thus warranting further investigations. Future research investigating various protocols of PRF, longer‐term outcomes, as well as PRF at the human histological level remains needed.

## 1 INTRODUCTION

Periodontal intrabony defects are a common feature of Stage III and IV periodontitis and are recognized as complexity factors in the current AAP/EFP classification of periodontal diseases^1^ based on the fact that they are associated with an increased risk of progression^2^ often requiring surgical intervention. Early pioneering work could demonstrate that intrabony defects have the potential for healing by regeneration employing barrier membranes (guided tissue regeneration [GTR]).^3^ Later, human histological evidence for regeneration became also available for decalcified‐freeze‐dried bone allograft (DFDBA),^4^ deproteinized bovine bone mineral (DBBM),^5^ enamel matrix derivative (EMD)^6^ and platelet‐derived growth factors.^7^

To date, based on a large number of RCTs published from 1990 to 2019, it is well established that regenerative surgical procedures using GTR, EMD, or bone grafts provide significant adjunctive clinical benefits to access flaps (open flap debridement [OFD]) alone and are recommended as treatment of choice for residual pockets with deep (≥3 mm) intrabony defects that are still present after Steps 1 and 2 of periodontal therapy.^8^, ^9^, ^10^

Yet to date, complete periodontal regeneration remains challenging and unpredictable.^11^, ^12^ Another strategy that was proposed several years ago for the regeneration of intrabony defects was the use of autogenous platelet concentrates (APCs). Platelet‐rich plasma (PRP) was the first generation platelet concentrate and its efficacy has been evaluated in a large number of randomized clinical trials between the years 2004 and 2019 and by systematic reviews (SRs).^13^, ^14^, ^15^, ^16^ The results indicated clinical benefits, when PRP was added to OFD alone, or to OFD + either DBBM or DFDBA versus OFD + xenografts/bone grafts alone (see Tables S1–S6 for representative studies).

The use of anticoagulants has since been shown to interfere with the angiogenic and regenerative responses mediated by platelets.^17^ For these reasons, a second‐generation platelet concentrate, termed platelet‐rich fibrin (PRF) has more recently been introduced in regenerative medicine and dentistry.^18^, ^19^, ^20^, ^21^, ^22^

More recent SRs on the use of APCs^23^, ^24^, ^25^, ^26^ have found a substantial increase in the number of RCTs that have evaluated the application of PRF to enhance clinical outcomes of OFD or of various modes of regenerative periodontal surgery. Some meta‐analyses seem to indicate a superiority of PRF over PRP with regard to probing pocket depth (PPD) reduction and clinical attachment level (CAL) gain; however, the only study with a direct comparison by Pradeep et al.^27^ reported similar PPD reduction, CAL gain, and bone fill (Tables S1–S6). Nevertheless, the authors concluded that PRF may seem a better treatment option because it is less time consuming and less technique sensitive.

Since PRF was first launched more than two decades ago in regenerative medicine, its use has gained widespread attention across many fields of medicine including periodontal regeneration. One of the advantages of PRF is that following centrifugation, it forms a fibrin‐dense clot with host platelets and leukocytes being entrapped favoring a more extended release of growth factors over time.^28^, ^29^

Several SRs with meta‐analysis, each including up to 21 randomized clinical trials, have already documented the use of PRF in the treatment of periodontal intrabony defects, indicating their potential efficacy in these clinical scenarios.^13^, ^15^, ^20^, ^30^, ^31^, ^32^, ^33^, ^34^

Likewise, the SR^8^ that served as evidence‐base for the application of regenerative surgery in the EFP S3‐Level Clinical Practice Guideline (CPG) for the treatment of periodontitis^9^, ^10^ stated that initial data seemed to support the use of PRF in addition to OFD, but also indicated the need for more studies assessing its clinical efficacy.

In the meantime, many more RCTs have been published, and therefore, it was the aim of this SR with meta‐analysis to evaluate the most up‐to‐date evidence on the efficacy of the use of PRF for the treatment of intrabony defects in comparison or addition to other established treatment options including bone grafts, barrier membranes, EMD membranes and other biomolecules used for periodontal regeneration.

## 2 MATERIALS AND METHODS

### 2.1 Protocol

This SR followed the recommendations of the PRISMA guidelines.^35^ The protocol for this SR was based on PRISMA‐P.^36^ There were no deviations from the initial protocol. The protocol of this SR was registered in the INPLASY database (number: 202430001).

### 2.2 Focused question

“In patients/teeth affected by intrabony periodontal defects, what is the efficacy of the use of PRF alone or associated with other biomaterials in regenerative periodontal surgery compared with access flap (OFD) alone or combined with other commonly used regenerative methods for the improvement of periodontal intrabony defects?”

### 2.3 Eligibility criteria and study selection process

The inclusion criteria were based on the PICOS strategy highlighted below.^37^ The search‐and‐screening process was conducted by two independent reviewing authors (R.J.M. and N.E.E.), commencing with the analysis of titles and abstracts. Furthermore, a hand search was performed and reviewed between authors. Next, full papers were selected for careful reading and matched with the eligibility criteria for future data extraction. The search concordance between the two reviewers was evaluated by Cohen's kappa (κ) test. Disagreements between the reviewing authors were resolved through careful discussion. Only studies meeting the following criteria were included:

- Population: Systemically healthy humans with periodontal intrabony defects.
- Intervention: Surgical treatment of bone defects through the use of PRF alone or in combination with other biomaterials with a follow‐up period of at least 6 months.
- Comparison: PRF versus OFD alone or in combination with other biomaterials.
- Outcomes: *Primary*: changes in PPD and CAL. *Secondary*: radiographic bone fill (RBF) and bone fill assessed through bone sounding or reentry (BS/BF).
- Study design: RCTs with a minimum of 10 patients.

### 2.4 Search strategy

**FIGURE 1:**
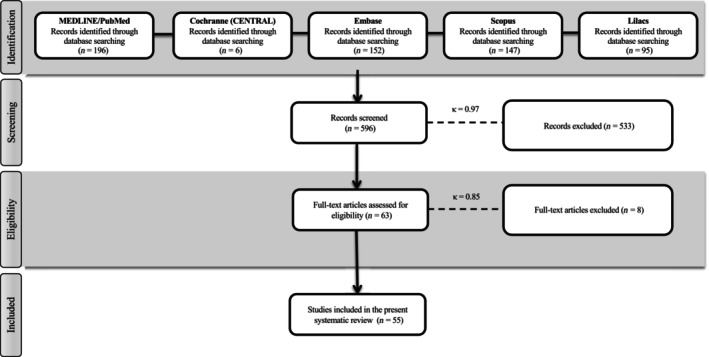
Search strategy.

PubMed/MEDLINE, the Cochrane Central Register of Controlled Trials, Scopus, Embase, and Lilacs were used to search for articles that were published before October 1, 2023 without other restrictions regarding date or language. A search of the gray literature using the Literature Report^38^ and OpenGrey^39^ databases was also conducted. Finally, the study reference lists were evaluated (cross‐referenced) to identify other studies for potential inclusion. The search strategy is described in Figure 1. Retrospective clinical studies, case reports, or animal studies as well as follow‐up of less than 6 months were excluded from the study.

### 2.5 Data synthesis

The study data were extracted by R.J.M., N.E.E., P.‐M.J.‐S., and S.J. and systematically reviewed by V.M. The following data, when available, were extracted from the included studies: authors, study design, follow‐up, number of treated intrabony defects, type of bone defects, number of subjects, age range, gender, number of smokers, surgical technique, mean difference (MD) in PPD, CAL, RBF, centrifugation system, volume of blood drawn, and centrifugation parameters.

### 2.6 Risk of bias within studies

Two review authors (P.‐M.J.‐S. and S.J.) assessed the methodological quality of included studies mainly using the risk of bias components shown to affect study outcomes, including method of randomization, allocation concealment, and blinding of examiners. The completeness of outcome reporting, selective outcome reporting, and other potential threats to validity were also assessed. Risk of bias was used in sensitivity analyses to test the robustness of the conclusions but was not used to exclude studies qualifying for the review.

The RoB 2 (RoB 2 tool from the *Cochrane Handbook for Systematic Reviews of Interventions*)^40^, ^41^ was used to analyze the risk of bias in RCTs. Each study was analyzed in relation to five domains: risk of bias arising from the randomization process, risk of bias due to deviations from the intended interventions, missing outcome data, risk of bias in the measurement of the outcome, and risk of bias in the selection of the reported research. A judgment about the risk of bias arising from each domain is proposed by an algorithm, based on answers to the signaling questions. Judgments can be “Low,” or “High” risk of bias, or can express “Some concerns.” The overall risk of bias is the least favorable assessment across the domains of bias, meaning: low risk, when all five domains of the study were judged as low risk; some concerns, when the study is judged as raising some concerns in at least one area; and high risk, when the study is judged to be at high risk in at least one domain We did not score performance bias of the surgeon, as it is impossible to blind the therapist performing various surgical treatments.

### 2.7 Risk of bias across studies

The different types of reporting bias that might have been present in this review were considered. If there were more than 10 studies included in a meta‐analysis, a funnel plot to detect possible publication bias should be created, and Egger's and Begg's tests applied.^42^

### 2.8 Statistical analysis

The continuous variables (PPD, CAL, and RBF) of the included studies were categorized into groups and subgroups and analyzed in a meta‐analysis through the software Review Manager (version 5.2.8, Copenhagen, Denmark, 2014).

The effects were estimated as a mean difference (MD) with 95% confidence interval (CI). The generic variation approach was adopted. Heterogeneity was assessed using the chi‐squared tests, with low heterogeneity considered for values ≤25%, moderate heterogeneity considered for values >25% but ≤50%, and high heterogeneity considered for values >50%.^42^ For the analyses, the random effect model was chosen due to the variation in available evidence (e.g., populations, follow‐up times, and settings). The statistical significance level of the meta‐analysis effect was set at *p* < 0.05.

## 3 RESULTS

### 3.1 Literature search

In total, 596 studies on intrabony were screened published until October 1, 2023, met the eligibility criteria, and were included in this SR. In total, 55 RCTs met the inclusion criteria (Table 1).^27^, ^43^, ^44^, ^45^, ^46^, ^47^, ^48^, ^49^, ^50^, ^51^, ^52^, ^53^, ^54^, ^55^, ^56^, ^57^, ^58^, ^59^, ^60^, ^61^, ^62^, ^63^, ^64^, ^65^, ^66^, ^67^, ^68^, ^69^, ^70^, ^71^, ^72^, ^73^, ^74^, ^75^, ^76^, ^77^, ^78^, ^79^, ^80^, ^81^, ^82^, ^83^, ^84^, ^85^, ^86^, ^87^, ^88^, ^89^, ^90^, ^91^, ^92^, ^93^, ^94^, ^95^, ^96^ The most highly researched centrifugation system utilized in 19 of 55 studies (35% of studies) was the Remi centrifuge (most commonly utilizing the R‐4C model). The IntraSpin/Hettich PC‐02 system was utilized in 4/55 studies (7% of studies). Shockingly, 19 of 55 studies (35% of studies) failed to report what system of centrifugation was utilized (later discussed). Of the 55 studies, 32/55 utilized a 3000 rpm for 10‐min protocol (48% of studies), whereas 4/55 studies utilized a 3000 rpm × 12‐min protocol and another 4/55 studies utilized a 2700 rpm for 12‐min protocol (7% of studies). Only three of 55 studies included smokers into their study.

**TABLE 1:** Main characteristics of the 55 randomized controlled trials (RCTs) included in the present study investigating intrabony defects treated with platelet‐rich fibrin (PRF).

Authors (year)	Study design follow‐up	No. of participants, Gender, Mean age	Groups (n defects)	Bone defect type	Smokers (No, Yes)	Conclusions
Group I (1): OFD vs. OFD/PRF						
Sharma and Pradeep (2011)^43^	RCT (parallel) 9 months	42 ♂24/♀18 35.3	C: 28, OFD T: 28, OFD + PRF	Three walls	No	There was greater PD reduction, CAL gain, and bone fill at sites treated with PRF with OFD compared with OFD alone
Thorat et al. (2011)^44^	RCT (parallel) 9 months	32 ♂20/♀12 30.7	C: 16, OFD T: 16, OFD + PRF	Two and three walls	No	There was greater reduction in PD, more CAL gain and greater intrabony defect fill at sites treated with PRF than the OFD alone
Pradeep et al. (2012)^27^	RCT (parallel) 9 months	33 ♂27/♀27 (total for three groups) 36.8	C: 17, OFD T: 16, OFD + PRF	Three walls	No	There were improvements in PD reduction, CAL gain, and BF at sites treated with PRF
Joseph et al. (2012)^45^	CT (split‐mouth) 12 months	15 ♂6/♀9 29.5	C: 15, OFD T: 15, OFD + PRF	Two and three walls	No	The use of PRF was more effective than OFD alone in the management of IBDs
Ajwani et al. (2015)^46^	RCT (split‐mouth) 9 months	20 ♂10/♀10 30.5	C: 20, OFD T: 20, OFD + PRF	Two and three walls	No	Adjunctive use of PRF with OFD significantly improves defect fill when compared to OFD alone
Pradeep et al. (2015)^47^	RCT (parallel) 9 months	60 ♂30/♀30 41	C: 30, OFD T: 30, OFD + PRF	Three walls	No	The study showed that the PRF was more effective than OFD alone in the management of IBDs
Chandradas et al. (2016)^48^	RCT (parallel) 9 months	24 ♂12/♀12 (total for three groups) 45.4	C: 12, OFD T: 12, OFD + PRF	Two and three walls	No	The study demonstrated that PRF improves clinical and radiological parameters compared with OFD alone in IBDs
Kanoriya et al. (2016)^49^	RCT (parallel) 9 months	60 ♂43/♀47 (total for three groups) 40.3	C: 30, OFD T: 30, OFD + PRF	Three walls	No	The use of PRF for IBD treatment showed better clinical parameter outcomes compared with OFD alone
Martande et al. (2016)^50^	RCT (parallel) 9 months	60 ♂48/♀48 (total for three groups) 37.6	C: 30, OFD T: 30, OFD + PRF	Three walls	No	PRF showed improvements in clinical parameters with a greater percentage radiographic defect depth reduction compared with OFD alone in treatment of IBDs
Pradeep et al. (2016)^51^	RCT (parallel) 9 months	60 ♂45/♀45 (total for three groups) 35	C: 30, OFD T: 30, OFD + PRF	Two and three walls	No	OFD with PRF results in significantly greater periodontal benefits compared with OFD alone
Bajaj et al. (2017)^52^	RCT (parallel) 9 months	17 ♂9/♀8 29.7	C: 27, OFD T: 27, OFD + PRF	Two and three walls	No	There was greater BF at sites treated with PRF with conventional OFD than conventional OFD alone
Chatterjee et al. (2017)^53^	RCT (parallel) 9 months	38 NR NR	C: 28, OFD T: 28, OFD + PRF	Three walls	No	A marked improvement in clinical parameters and radiographic outcomes were observed in the PRF group when compared to OFD
Patel et al. (2017)^54^	RCT (split‐mouth) 12 months	13 ♂4/♀9 44	C: 13, OFD T: 13, OFD + PRF	Two and three walls	No	The adjunctive use of PRF to conventional OFD may be potentially used in the treatment of IBDs
Pradeep et al. (2017)^55^	RCT (parallel) 9 months	37 ♂34/♀28 (total for three groups) 39.7	C: 18, OFD T: 19, OFD + PRF	Three walls	No	Treatment of IBD with PRF results in significant improvements of clinical parameters compared with baseline
Thorat et al. (2017)^56^	RCT (split‐mouth) 12 months	15 ♂7/♀8 25	C: 15, OFD T: 15, OFD + PRF	Three walls	No	Use of PRF significantly enhances the clinical and radiographic outcomes of OFD in the treatment of IBDs
Yajamanya et al. (2017)^57^	RCT (parallel) 9 months	32 NR NR	C: 28, OFD T: 28, OFD + PRF	Two and three walls	No	This study shows marked improvements in the clinical parameters and radiographic outcomes with PRF to treat periodontal IBDs as compared to OFD alone
Bahammam and Attia (2021)^58^	RCT (parallel) 6 months	30 ♂17/♀13 39.7	C: 15, OFD T: 15, OFD + PRF	NR	No	The use of PRF improved PD and defect fill compared with OFD alone
Pham et al. (2021)^59^	RCT (split‐mouth) 12 months	20 ♂22/♀8 48	C: 10, OFD T: 10, OFD + PRF	Two and three walls	No	Compared with OFD alone, PRF significantly improved clinical and radiographic parameters
Abdulrahman et al. (2022)^60^	RCT (parallel) 9 months	22 ♂4/♀18 36	C: 11, OFD T: 11, OFD + PRF	Two and three walls	No	The use of low‐speed PRF in conjunction with OFD improved CAL and PD post‐surgically
Mubarak et al. (2023)^61^	RCT (parallel) 6 months	30 ♂12/♀18 38.75	C: 10, OFD T: 10, OFD + PRF	Two or three walls	No	Compared with OFD alone, PRF significantly improved clinical and radiographic parameters
Ugale et al. (2023)^62^	RCT (parallel) 6 months	46 ♂14/♀32 36.30	C: 23, OFD T: 23, OFD + PRF	Two or three walls	No	Only mild yet nonsignificant differences were reported with the addition of PRF
Group I (2): OFD vs. OFD/T‐PRF						
Ustaoğlu et al. (2020)^63^	RCT (parallel) 9 months	30 ♂23/♀22 (all three groups) 40	C: 15, OFD T: 15, OFD + T‐PRF	Two or three walls	No	The PRF group showed significant improvement in clinical parameters compared with the OFD alone (control group) at 9 months
Group II: Comparing PRF protocols						
(3): OFD/PRF vs. OFD/T‐PRF						
Chatterjee et al. (2017)^53^	RCT (parallel) 9 months	38 NR NR	C: 28, OFD + PRF T: 28, OFD + T‐PRF	Three walls	No	No statistically significant differences were observed between the PRF and T‐PRF groups
Gummaluri et al. (2020)^64^	RCT (parallel) 9 months	26 ♂15/♀11 33.3	C: 17, OFD + L‐PRF T: 17, OFD + T‐PRF	Three walls	No	T‐PRF seems to be a better alternative to L‐PRF in the treatment of intrabony defects
Group III: Comparative studies to PRF						
(4) OFD/PRP vs. OFD/PRF						
Pradeep et al. (2012)^27^	RCT (parallel) 9 months	33 ♂27/♀27 (total for three groups) 36.8	C1: 17, OFD + PRP T: 16, OFD + PRF	Three walls	No	There was similar PD reduction, CAL gain, and BF at sites treated with PRF or PRP with conventional OFD
(5) OFD/BG/PGRF vs. OFD/BG/PRF						
Gamal et al. (2016)^65^	RCT (parallel) 9 months	20 ♂15/♀5 42	C: 10, OFD + Xenograft + PRGF T: 10, OFD + Xenograft + PRF	Two and three walls	No	No significant differences were found between the test and control group in terms of clinical periodontal parameters and growth factor levels at treated sites
(6) OFD/EMD vs. OFD/PRF						
Gupta et al. (2014)^66^	RCT (parallel) 6 months	30 ♂15/♀15 NR	C: 22, OFD + EMD T: 22, OFD + PRF	Three walls	No	Both EMD and PRF were effective in the regeneration of IBDs
Csifo‐Nagy et al. (2021)^67^	RCT (parallel) 6 months	18 ♂9/♀9 55.5	C: 15, OFD + EMD T: 15, OFD + PRF	Two and three walls	No	Platelet‐rich fibrin seems to be as clinically effective as EMD during surgical treatment of IBDs
(7) BG + EMD vs. BG + PRF						
Paolantonio et al. (2020)^68^	RCT (parallel) 12 months	44 ♂15/♀29 53	C: 22, BG + EMD T: 22, BG + PRF	“unfavorable” (predominantly one‐ combined one‐ to two‐ and two‐wall defects/craters, circumferential defects (at least three surfaces involved), or teeth with a wide defect angle (≥36))	No	PRF + BG treatment of noncontained intrabony defects produces noninferior results in terms of CAL gain, PPD reduction, and radiographic bone level gain in comparison with the EMD + BG combination
(8) BM vs. PRF						
Ustaoğlu et al. (2020)^63^	RCT (parallel) 9 months	30 (for all three groups) 40	T: 15, OFD + BM T: 15, OFD + T‐PRF	Two or three walls	No	T‐PRF may give similar successful results as GTR in the treatment of IBDs with endo‐perio lesions
Pham et al. (2021)^59^	RCT (split‐mouth) 12 months	20 ♂22/♀8 48	C: 10, OFD + BM T: 10, OFD + PRF	Two and three walls	No	Compared with GTR, PRF yielded comparable treatment outcomes in terms of improvements in clinical and radiographic parameters
(9) BG + BM vs. BG + PRF						
Aggour and Abd El‐Hady (2017)^69^	RCT (split‐mouth) 6 months	16 ♂10/♀6 24	T1: 16, OFD + Auto/Xeno T2: 16, OFD + Auto/Xeno + PRF	Two and three walls	No	Platelet‐rich fi brin has shown favorable results that are comparable to CM for treatment of intrabony periodontal defects in patients with GAgP
Rexhepi et al. (2021)^70^	RCT (parallel) 12 months	62 ♂26/♀36 55	C: 31, BG + BM T: 31, BG + PRF	“unfavorable” (predominantly one‐ combined one‐ to two‐ and two‐wall defects/craters, circumferential defects (at least three surfaces involved) or teeth with a wide defect angle (≥36))	No	The L‐PRF + BG treatment of unfavorable intrabony defects showed better outcomes for CAL gain, and radiographic bone level gain, while no difference in PPD reduction when compared to the BG + BM treatment
(10) BG vs. PRF						
Mathur et al. (2015)^71^	RCT (parallel) 6 months	25 ♂14/♀11 39.7	C: 19, OFD + ABG T: 19, OFD + PRF	Two and three walls	No	The use of either PRF or ABG were effective in the treatment of IBDs
Chadwick et al. (2016)^72^	RCT (parallel) 6 months	36 ♂20/♀16 54.9	C: 19, OFD + DFDBA T: 17, OFD + PRF	Two and three walls	Yes	Treatment of IBDs with either DFDBA or PRF resulted in a significant gain in CAL and BF after 6 months of healing, with no significant difference
Galav et al. (2016)^73^	RCT (split‐mouth) 9 months	20 NR 45	C: 20, OFD + ABG T: 20, OFD + PRF	Two and three walls	No	Both ABG and PRF can be used predictably to reconstruct lost periodontal structures with ABG leading to better outcomes
Agarwal et al. (2017)^74^	RCT (parallel) 6 months	26 NR NR	C: 12, OFD + NovaBone T: 14, OFD + PRF	Two and three walls	No	Both modalities led to similar outcomes
Yajamanya et al. (2017)^57^	RCT (parallel) 9 months	32 NR NR	C: 28, OFD + BioGlass T: 28, OFD + PRF	Two and three walls	No	This study shows marked improvements in the clinical parameters and radiographic outcomes with both BioGlass and PRF to treat periodontal IBDs as compared to OFD alone
Bahammam and Attia (2021)^58^	RCT (parallel) 6 months	30 ♂17/♀13	C: 15, OFD + HA T: 15, OFD + HA + PRF	NR	No	The combination use of PRF with nano‐HA was better in defect fill
Group IV: Addition of PRF to treatment groups						
(11) BG vs. BG + PRF						
Bansal and Bharti (2013)^75^	RCT (split‐mouth) 6 months	10 NR NR	C: 10, OFD + DFDBA T: 10, OFD + DFDBA + PRF	NR	NR	There was a significantly greater PD reduction and CAL when PRF was added to DFDBA
Elgendy and Abo Shady (2015)^76^	RCT (split‐mouth) 6 months	20 NR 44	C: 20, OFD + HA T: 20, OFD + HA + PRF	NR	Yes	Both treatment groups showed a significant PD reduction and CAL gain 6 months after surgery. However, there was a significantly greater PD reduction and CAL gain when PRF was added to BG
Agarwal et al. (2016)^77^	RCT (split‐mouth) 12 months	30 ♂15/♀15 52	C: 30, OFD + DFDBA T: 30, OFD + DFDBA/PRF	Two and three walls	No	The combination of PRF and DFDBA is more effective than DFDBA alone
Gamal et al. (2016)^65^	RCT (parallel) 9 months	19 ♂13/♀6 39	C: 9, OFD + Xenograft T: 10, OFD + Xenograft + PRF	Two and three walls	No	No significant differences were found between the test and control group in terms of clinical periodontal parameters and growth factor levels at treated sites
Agarwal et al. (2017)^74^	RCT (parallel) 6 months	26 NR NR	C: 12, OFD + NovaBone T2: 14, OFD + Novabone + PRF	Two and three walls	No	Combination of CPS and PRF showed a significant improvement in PD reduction, CAL gain, and bone fill
Naqvi et al. (2017)^78^	RCT (split‐mouth) 9 months	10 ♂7/♀3 NR	C: 10, OFD + BioG T: 10, OFD + BioG + PRF	Two and three walls	No	The results of this study showed both the groups BioG putty alone and the combination of PRF and BioG putty are effective in the treatment of IBDs
Sezgin et al. (2017)^79^	RCT (split‐mouth) 6 months	15 ♂8/♀7 NR	C: 15, OFD + ABBM T: 15, OFD + ABBM + PRF	Two and three walls	No	The results of this study indicate that both therapies are effective in the treatment of intrabony defects
Bodhare et al. (2019)^80^	RCT (split‐mouth) 6 months	20 ♂11/♀9 35.9	C: 20, OFD + BioGlass T: 20, OFD + BioGlass + PRF	Two and three walls	No	BioGlass when used in combination with PRF is found to be more effective in gain in CAL, reduction in PD, and achieving greater bone fill as compared to treatment with BG alone
Saravanan et al. (2019)^81^	RCT (split‐mouth) 6 months	15 NR 25–50	C: 15, OFD + Perioglass T: 15, OFD + Perioglass + PRF	NR	No	The incorporation of PRF with synthetic bone graft (Perioglass) produces effective and rapid periodontal regeneration with improved healing in intrabony osseous defects
Atchuta et al. (2020)^82^	RCT (parallel) 6 months	26 NR 25–55	C: 13, DFDBA T: 13, DFDBA + PRF	Two and three walls	No	Combination of DFDBA and PRF improved the clinical and radiographic parameters compared with DFDBA alone
Goyal et al. (2020)^83^	RCT (split‐mouth) 6 months	12 ♂10/♀2 40.0	C: 12, OFD + xenograft T: 12, OFD + xeno + PRF	Three walls	No	Combination of xenograft and PRF had better regeneration potential for the management of intrabony defects
Bahammam and Attia (2021)^58^	RCT (parallel) 6 months	30 ♂16/♀14 38.8	C: 15, OFD + HA T: 15, OFD + HA + PRF	NR	No	The combination use of PRF with nano‐HA was better in defect fill primarily
Hazari et al. (2021)^84^	RCT (parallel) 6 months	20 NR 25–55	C: 10, OFD + NovaBone T: 10, OFD + NovaBone + PRF	Three walls	No	Statistical more significant difference (*p* < 0.05) in PD, and CAL was observed when Novabone putty was combined with PRF vs. Novabone alone
Pavani et al. (2021)^85^	RCT (parallel) 6 months	20 NR NR	C: 10, OFD + B‐TCP T: 10, OFD + B‐TCP + PRF	Three walls	Yes	Bone fill achieved in β‐TCP with PRF was more compared with β‐TCP alone and OFD at 6‐month follow‐up
Alshoiby et al. (2023)^86^	RCT (parallel) 3, 6, and 9 months	20 ♂7/♀13 31.3	C: 10, OFD + DFDBA T: 10, OFD + DFDBA + iPRF	Two and three walls	No	Addition of I‐PRF to DFDBA does not appear to significantly enhance the DFDBA's reparative/regenerative outcomes
Baghele et al. (2023)^87^	RCT (split‐mouth) 6 months	26 ♂25/♀1 40.0	C: 21, OFD + BCP T: 21, OFD + BCP + PRF	Three walls	No	The combination use of BCP with PRF resulted in statistically significant improvement in CAL and PPD and radiographic bone gain
Chaudhary et al. (2023)^88^	RCT (parallel) 6 and 9 months	34 ♂18/♀16 20–55	C: 17, OFD + HA T: 17, OFD + HA + i‐PRF	Three walls	No	No significant differences were reported when combining i‐PRF with HA
(12) GTR vs. GTR + PRF						
Panda et al. (2016)^89^	RCT (split‐mouth) 9 months	18 ♂10/♀8 38.1	C: 18, OFD + BM T: 18, OFD + BM + PRF	Three walls	No	The addition of PRF to GTR led to better outcomes in all investigated parameters
(13) EMD vs. EMD + PRF						
Aydemir Turkal et al. (2016)^90^	RCT (split‐mouth) 6 months	28 ♂14/♀14 38.5	C: 24, OFD + EMD T: 25, OFD + EMD + PRF	One, two and three walls	No	Addition of PRF did not improve the clinical and radiographic outcomes
(14) BG + BM vs. BG + BM + PRF						
Liu et al. (2021)^91^	RCT (split‐mouth) 12 and 24 months	14 ♂4/♀10 36	C: 14, BG + BM T: 14, BG + BM + PRF	NR	No	Compared with BG + BM, the combination of BM + BG + PRF is more effective clinically, and results in better clinical outcomes
Group V: Addition of biomaterial/biomolecule to PRF						
(15) PRF vs. PRF + BG						
Lekovic et al. (2012)^92^	RCT (split‐mouth) 6 months	17 ♂6/♀11 44	C: 17, OFD + PRF T: 17, OFD + BPBM + PRF	Two and three walls	Yes	The adjunctive use of BPBM in combination with BPBM displayed a significantly greater resolution of intrabony defects in periodontal regenerative therapy
Chandradas et al. (2016)^48^	RCT (parallel) 9 months	24 18/18 (total for three groups) 43.1	C: 12, OFD + PRF T: 12, OFD + PRF + DBM	Two and three walls	No	Addition of DBM enhances the effects of PRF in RAL gain and radiographic defect fill
Agarwal et al. (2017)^74^	RCT (parallel) 6 months	26 NR NR	C: 12, OFD + NovaBone T: 14, OFD + PRF + Novabone	Two and three walls	No	Combination of PRF and CPS putty showed a significant improvement in PD reduction, CAL gain, and bone fill
Pradeep et al. (2017)^55^	RCT (parallel) 9 months	39 ♂34/♀28 (total for three groups)	C: 19, OFD + PRF T: 20, OFD + PRF + HA	Three walls	No	HA when added to PRF increases the regenerative effects observed with PRF in the treatment of human three‐wall intrabony defects
Bahammam and Attia (2021)^58^	RCT (parallel) 6 months	30 ♂15/♀15 NR	C: 15, OFD + PRF T: 15, OFD + PRF + HA	NR	No	The combination use of PRF with nano‐HA was better in regenerative periodontal therapy to manage periodontal IBDs
Thetay et al. (2021)^93^	RCT (parallel) 9 months	60 ♂33/♀27 NR	C: 30, OFD + PRF T: 30, OFD + PRF + HA	Three walls	No	Treatment of IBDs with PRF + HA showed a significant improvement in bone fill but not PD or CAL
(16) PRF vs. PRF + AA						
Elbehwashy et al. (2021)^94^	RCT (parallel) 6 months	20 ♂3/♀17 30	C: 10, PRF T: 10, PRF + AA (ascorbic acid)	Two and three walls	No	PRF, with or without AA, could significantly improve periodontal parameters. Supplementing PRF with AA could additionally augment radiographic linear defect fill
(17) PRF vs. PRF + Metformin						
Pradeep et al. (2015)^47^	RCT (parallel) 9 months	60 ♂30/♀30 41	C: 30, OFD + PRF T: 30, OFD + PRF + 1% MF	Three walls	No	The study showed that the PRF + 1% MF group was more effective than MF, PRF, or OFD alone in the management of IBDs
(18) PRF vs. PRF + Bisphosphonates						
Kanoriya et al. (2016)^49^	RCT (parallel) 9 months	60 ♂43/♀47 (total for three groups) 40.3	C: 30, OFD + PRF T: 30, OFD + PRF/1% ALN	Three walls	No	Combined approach therapy of PRF + 1% ALN for IBDs treatment showed better clinical parameter outcomes compared with PRF and OFD alone
(19) PRF vs. PRF + Statins						
Martande et al. (2016)^50^	RCT (parallel) 9 months	60 ♂48/♀48 (total for three groups) 37.6	C: 30, OFD + PRF T: 30, OFD + PRF + 1.2% ATV	Three walls	No	PRF + 1.2% ATV showed similar improvements in clinical parameters with a greater percentage radiographic defect depth reduction compared with PRF alone in treatment of IBDs
Pradeep et al. (2016)^51^	RCT (parallel) 9 months	60 ♂45/♀45 (total for three groups) 35	C: 30, OFD + PRF T: 30, OFD + PRF + 1.2% RSV	Two and three walls	No	OFD with RSV (1.2%) and PRF results in significantly greater periodontal benefits compared with OFD alone or with PRF
(20) BG + PRF vs. BG + PRF + Statins						
Gautam et al. (2022)^95^	RCT (parallel) 9 months	39 ♂16/♀23 37	C: 13, OFD T1: 13, OFD + BG + PRF T2:13, OFD + BG + PRF + Rosuvastatin	Two and three walls	No	Addition of Rosuvastatin gel to PRF and BG improved clinical outcomes and defect fill
(21) PRF vs. PRF + LLLT						
Thalaimalai et al. (2020)^96^	RCT (parallel) 6 months	30 NR NR	C: 15, SPPF + PRF T: 15, SPPT + PRF + LLLT	Two and three walls	No	Only mild nonsignificant improvements were observed

Clinical and radiographic outcomes							
Authors (year)	Mean difference in PPD between baseline and final follow‐up (mm)	Mean difference in CAL between baseline and final follow‐up (mm)	Mean difference in BS‐BF between baseline and final follow‐up (mm)	Mean difference in RBF between baseline and final follow‐up (mm/%)	Methods for PRF preparation		
					Centrifugation system	Volume of blood drawn	Centrifugation parameters speed (rpm) × time (min)
Group I: OFD vs. PRF							
(1) OFD vs. PRF							
Sharma and Pradeep (2011)	3.21 ± 1.64 (C) 4.55 ± 1.87 (T)	2.77 ± 1.44 (C) 3.31 ± 1.76 (T)		0.09 ± 0.11 (C) 2.50 ± 0.78 (T)	R‐4C (REMI, Mumbai, India)	10 mL	3000 × 10
Thorat et al. (2011)	3.56 ± 1.09 (C) 4.69 ± 1.45 (T)	2.13 ± 1.71 (C) 4.13 ± 1.63 (T)		1.24 ± 0.69 (C) 2.12 ± 0.69 (T)	NR	10 mL	2700 × 12
Pradeep et al. (2012)	2.97 ± 0.93 (C) 3.77 ± 1.19 (T)	2.83 ± 0.91 (C) 3.17 ± 1.29 (T)		0.13 ± 1.46 (C) 2.80 ± 0.89 (T)	R‐4C (REMI, Mumbai, India)	10 mL	3000 × 10
Joseph et al. (2012)	2.40 ± 0.63 (C) 4.67 ± 0.90 (T)	1.40 ± 1.06 (C) 4.73 ± 0.88 (T)		0.64 ± 0.50 (C) 1.93 ± 1.07 (T)	KW‐70 (Almicro™ Instruments, Haryana, India)	10 mL	3000 × 10
Ajwani et al. (2015)	1.60 ± 0.67(C) 1.90 ± 0.92 (T)	1.30 ± 1.84 (C) 1.80 ± 1.39 (T)		0.80 ± 1.23 (C) 1.45 ± 0.51 (T)	R‐4C (REMI, Mumbai, India)	10 mL	3000 × 10
Pradeep et al. (2015)	3.00 ± 0.18 (C) 4.00 ± 0.18 (T)	2.96 ± 0.18 (C) 4.03 ± 0.18 (T)		0.49 ± 0.27 (C) 2.53 ± 0.30 (T)	R‐4C (REMI, Mumbai, India)	10 mL	3000 × 10
Chandradas et al. (2016)	3.00 ± 1.21 (C) 3.82 ± 0.75 (T)	2.25 ± 0.62 (C) 3.27 ± 0.65 (T)		1.22 ± 0.62 (C) 2.30 ± 0.83 (T)	R‐4C (REMI, Mumbai, India)	10 mL	3000 × 12
Kanoriya et al. (2016)	2.86 ± 0.68 (C) 3.70 ± 0.91 (T)	3.03 ± 0.18 (C) 4.20 ± 0.66 (T)		0.38 ± 0.26 (C) 2.42 ± 0.21 (T)	R‐4C (REMI, Mumbai, India)	10 mL	3000 × 10
Martande et al. (2016)	2.76 ± 1.43 (C) 3.76 ± 1.12 (T)	2.50 ± 1.33 (C) 3.40 ± 1.13 (T)		0.27 ± 0.19 (C) 2.46 ± 0.33 (T)	R‐4C (REMI, Mumbai, India)	5 mL	3000 × 12 to 14
Pradeep et al. (2016)	3.10 ± 0.30 (C) 4.03 ± 0.18 (T)	2.47 ± 0.77 (C) 3.30 ± 0.65 (T)		1.43 ± 0.50 (C) 3.17 ± 0.65 (T)	R‐4C (REMI, Mumbai, India)	10 mL	3000 × 10
Bajaj et al. (2017)	2.14 ± 1.26 (C) 3.14 ± 1.26 (T)	1.59 ± 1.01 (C) 2.66 ± 1.07 (T)		0.84 ± 0.99 (C) 2.24 ± 0.66 (T)	R‐4C (REMI, Mumbai, India)	10 mL	3000 × 10
Chatterjee et al. (2017)	3.68 ± 0.72 (C) 5.46 ± 1.04 (T)	4.14 ± 0.76 (C) 6.57 ± 1.45 (T)			NR	5 mL	373.3 (g) × 10
Patel et al. (2017)	2.40 ± 0.84 (C) 4.20 ± 1.69 (T)	2.10 ± 0.74 (C) 3.70 ± 0.67 (T)			REMI‐8C (REMI, Mumbai, India)	10 mL	3000 × 10
Pradeep et al. (2017)	2.97 ± 0.93 (C) 3.90 ± 1.09 (T)	2.67 ± 1.09 (C) 3.03 ± 1.16 (T)		0.93 ± 0.83 (C) 3.20 ± 0.89 (T)	R‐4C (REMI, Mumbai, India)	10 mL	3000 × 10
Thorat et al. (2017)	1.50 ± 0.34 (C) 4.00 ± 0.63 (T)	0.33 ± 1.21 (C) 4.00 ± 0.63 (T)		1.67 ± 1.33 (C) 3.09 ± 1.16 (T)	R‐4C (REMI, Mumbai, India)	10 mL	3000 × 12
Yajamanya et al. (2017)	3.68 ± 0.72 (C) 6.11 ± 0.92 (T)	4.14 ± 0.76 (C) 6.74 ± 1.55 (T)			NR	10 mL	3000 × 10
Bahammam and Attia (2021)	0.90 ± 1.10 (C) 2.7 ± 0.89 (T)	1.0 ± 1.13 (C) 1.17 ± 1.21 (T)		1.1 ± 0.57 (C) 2.2 ± 0. 09 (T)	NR	5 mL	3000 × 10
Pham et al. (2021)	3.37 ± 1.00 (C) 4.80 ± 0.71 (T)	3.37 ± 1.22 (C) 5.00 ± 0.46 (T)		2.23 ± 0.86 (C) 4.13 ± 0.51 (T)	(Hettich EBA 200, Germany)	3 × 10 mL	2500 × 15
Abdulrahman et al. (2022)	2.18 ± 1.17 (C) 3.73 ± 1.19 (T)	2.45 ± 0.93 (C) 3.55 ± 1.37 (T)		1.32 ± 1.21 (C) 2.09 ± 0.73 (T)	VE‐4000 (Velab, Texas, USA)	10 mL	1300 × 8
Mubarak et al. (2023)	3.80 ± 0.86 (C) 3.80 ± 0.98 (T)	3.10 ± 0.87(C) 3.00 ± 0.85 (T)		0.43 ± 0.54 (C) 0.80 ± 0.62 (T)	Intraspin (Boca Raton, Florida, USA)	10 mL	2700 × 12
Ugale et al. (2023)	2.91 ± 1.41(C) 3.39 ± 1.76 (T)	3.44 ± 1.30 (C) 2.91 ± 1.46 (T)		0.31 ± 1.12 (C) 1.57 ± 0.59 (T)	REMI Lab	10 mL	3000 × 10
(2) OFD vs. T‐PRF							
Ustaoğlu et al. (2020)	3.36 ± 1.12 (C) 4.69 ± 1.34 (T)	3.30 ± 1.17 (C) 4.19 ± 1.05 (T)		0.90 ± 0.80 (C) 2.97 ± 0.77 (T)	NR	NR	2800 × 12
Group II: Comparing PRF protocols							
(3) L‐PRF vs. T‐PRF							
Chatterjee et al. (2017)	5.46 ± 1.04 (C) 6.25 ± 1.11 (T)	6.57 ± 1.45 (C) 6.74 ± 1.55 (T)			NR	5 mL	3000 × 10
Gummaluri et al. (2020)	4.88 ± 0.92 (C) 5.24 ± 1.30 (T)	4.24 ± 1.25 (C) 4.94 ± 1.43 (T)		1.68 ± 3.28 (C) 2.05 ± 2.56 (T)	R‐8C (REMI, Mumbai, India)	10 mL	L‐PRF = 2800 × 12 T‐PRF = 3500 × 15
Group III: Comparative studies to PRF							
(4) PRP vs. PRF							
Pradeep et al. (2012)	3.77 ± 1.07 (C) 3.77 ± 1.19 (T)	2.93 ± 1.08 (C) 3.17 ± 1.29 (T)		2.70 ± 0.79 (C) 2.80 ± 0.89 (T)	R‐4C (REMI, Mumbai, India)	10 mL	3000 × 10
(5) BG + PRGF vs. BG + PRF							
Gamal et al. (2016)	4.3 ± 0.26 (C) 3.6 ± 0.35 (T)	2.0 ± 0.44 (C) 1.2 ± 0.26 (T)		2.1 ± 0.4 (C) 1.7 ± 0.3 (T)	NR	NR	NR
(6) EMD vs. PRF							
Gupta et al. (2014)	1.80 ± 0.56 (C) 1.80 ± 0.77 (T)	2.00 ± 0.54 (C) 1.87 ± 0.91 (T)		2.08 ± 0.77 (C) 1.67 ± 1.17 (T)	NR (REMI, Mumbai, India)	10 mL	3000 × 12
Csifó‐Nagy et al. (2021)	3.46 ± 1.30 (C) 3.6 ± 1.68 (T)	2.6 ± 1.18 (C) 2.33 ± 1.59(T)	3.8 ± 1.56 (C) 3.93 ± 1.98 (T)		Duo Quattro (Process, Nice, France)	10 mL	1300 × 8
(7) BG + EMD vs. BG + PRF							
Paolantonio et al. (2020)	3.96 ± 1.04 (C) 4.21 ± 1.10 (T)	3.29 ± 0.85 (C) 3.43 ± 0.74 (T)		2.68 ± 1.06 (C) 2.93 ± 0.72 (T)	IntraSpin (Intra‐Lock System Europa SpA, Salerno, Italy)	3 × 10 mL	3000 × 10
(8) BM vs. PRF							
Ustaoğlu et al. (2020)	5.67 ± 1.21 (C) 4.69 ± 1.34 (T)	5.50 ± 1.53 (C) 4.19 ± 1.05 (T)		3.85 ± 1.16 (C) 2.97 ± 0.77 (T)	NR	NR	2800 × 12
Pham et al. (2021)	4.63 ± 0.67 (C) 4.80 ± 0.71 (T)	4.53 ± 0.57 (C) 5.00 ± 0.46 (T)		3.97 ± 0.72 (C) 4.13 ± 0.51 (T)	(Hettich EBA 200, Germany)	3 × 10 mL	2500 × 15
(9) BG + BM vs. BG + PRF							
Aggour and Abd El‐Hady (2017)	3.92 ± 0.82 (C) 3.92 ± 0.75 (T)	4.04 ± 0.89 (C) 4.50 ± 0.92 (T)		3.13 ± 1.64 (C) 3.50 ± 1.67 (T)	Tabletop centrifuge (Shanghai Medical Instruments, China)	10 mL	400 g × 10
Rexhepi et al. (2021)	4.81 ± 1.33 (C) 4.26 ± 1.18 (T)	2.87 ± 1.28 (C) 3.48 ± 1.26 (T)		2.19 ± 1.44 (C) 2.77 ± 1.43 (T)	IntraSpin (Intra‐Lock System Europa, Salerno, Italy)	3 × 10 mL	3000 × 10
(10) BG vs. PRF							
Mathur et al. (2015)	2.40 ± 1.06 (C) 2.67 ± 1.29 (T)	2.67 ± 1.63 (C) 2.53 ± 1.06 (T)	2.66 ± 1.84 (C) 2.93 ± 1.79 (T)	1.76 ± 0.83 (C) 3.94 ± 3.56 (T)	R‐4C (REMI, Mumbai, India)	NR	3000 × 10
Chadwick et al. (2016)	2.00 ± 1.37 (C) 2.12 ± 1.41 (T)	1.16 ± 1.33 (C) 1.03 ± 0.86 (T)	1.53 ± 1.64 (C) 1.35 ± 1.60 (T)	1.14 ± 0.88 (C) 1.10 ± 1.01 (T)	Centrifuge 1310 (Salvin Dental Specialties, Charlotte, NC)	10 mL	3000 × 10
Galav et al. (2016)	4.80 ± 0.57 (C) 4.10 ± 0.63 (T)	4.50 ± 0.61(C) 3.90 ± 0.57 (T)	1.79 ± 0.28 (C) 1.17 ± 0.34 (T)	3.20 ± 0.43 (C) 2.40 ± 0.57 (T)	NR	10 mL	3000 × 10
Agarwal et al. (2017)	3.50 ± 1.73 (C) 3.50 ± 1.95 (T)	2.50 ± 2.78 (C) 2.00 ± 2.25 (T)		3.00 ± 3.03 (C) 1.50 ± 2.14 (T)	NR	NR	NR
Yajamanya et al. (2017)	5.57 ± 1.10 (C) 6.11 ± 0.92 (T)	6.57 ± 1.45 (C) 6.74 ± 1.55 (T)			NR	10 mL	3000 × 10
Bahammam and Attia (2021)	2.4 ± 1.17 (C) 2.7 ± 0.89 (T)	1.7 ± 1.03 (C) 1.17 ± 1.21 (T)		1.49 ± 0.65 (C) 2.2 ± 0. 09 (T)	NR	5 mL	3000 × 10
Group IV: Addition of PRF to treatment groups							
(11) BG vs. BG + PRF							
Bansal and Bharti (2013)	3.10 ± 0.74 (C) 4.00 ± 0.82 (T)	2.30 ± 0.70 (C) 3.40 ± 0.60 (T)		1.93 ± 1.21 (C) 2.13 ± 1.28 (T)	NR	10 mL	3000 × 10
Elgendy and Abo Shady (2015)	3.30 ± 0.64 (C) 3.33 ± 0.74 (T)	3.50 ± 0.47 (C) 3.55 ± 0.58 (T)			NR	10 mL	3000 × 10
Agarwal et al. (2016)	3.60 ± 0.51 (C) 4.15 ± 0.84 (T)	2.61 ± 0.68 (C) 3.73 ± 0.74 (T)		2.49 ± 0.64 (C) 3.50 ± 0.67 (T)	NR	10 mL	400 g × 12
Gamal et al. (2016)	3.8 ± 0.3 (C) 3.6 ± 0.35 (T)	1.8 ± 0.35 (C) 1.2 ± 0.26 (T)		1.6 ± 0.36 (C) 1.7 ± 0.3 (T)	NR	NR	NR
Agarwal et al. (2017)	3.50 ± 1.73 (C) 4.50 ± 2.83 (T)	2.50 ± 2.78 (C) 3.00 ± 3.28 (T)		3.00 ± 3.03 (C) 2.50 ± 2.14 (T)	NR	NR	NR
Naqvi et al. (2017)	3.15 ± 1.06 (C) 3.20 ± 2.30 (T)	3.15 ± 1.06 (C) 4.10 ± 1.73 (T)		5.70 ± 1.64 (C) 7.10 ± 1.37 (T)	NR	10 mL	3000 × 10
Sezgin et al. (2017)	4.21 ± 1.21 (C) 4.93 ± 1.22 (T)	3.27 ± 1.34 (C) 4.47 ± 1.60 (T)		1.98 ± 0.80 (C) 2.55 ± 1.15 (T)	PC‐02 (Process, Nice, France)	10 mL	2700 × 12
Bodhare et al. (2019)	5.65 ± 1.66 (C) 5.75 ± 1.16 (T)	4.20 ± 1.70 (C) 5.05 ± 1.09 (T)		2.56 ± 0.95 (C) 3.51 ± 1.17 (T)	REMI, Mumbai, India	10 mL	3000 × 10
Saravanan et al. (2019)	2.00 ± 0.24 (C) 3.93 ± 0.23 (T)	2.60 ± 0.29 (C) 4.93 ± 0.34 (T)		0.79 ± 0.07 (C) 1.24 ± 0.04 (T)	NR	10 mL	3000 × 10
Atchuta et al. (2020)	3.56 ± 1.56 (C) 3.60 ± 1.42 (T)	3.50 ± 1.48 (C) 3.40 ± 1.65 (T)		5.39 ± 2.07 (C) 5.35 ± 2.05 (T)	REMI, Mumbai, India	10 mL	3000 × 10
Goyal et al. (2020)	4.08 ± 0.79 (C) 4.58 ± 0.67 (T)	4.00 ± 0.85 (C) 4.91 ± 0.67 (T)		1.42 ± 0.67 (C) 2.15 ± 0.72 (T)	REMI, Mumbai, India	10 mL	2700 × 12
Bahammam and Attia (2021)	2.4 ± 1.17 (C) 3. 0 ± 0.94 (T)	1.7 ± 1.03 (C) 2.1 ± 1.04 (T)		1.49 ± 0.65 (C) 2.31 ± 1.50 (T)	NR	5 mL	3000 × 10
Hazari et al. (2021)	1.50 ± 0.53 (C) 2.60 ± 0.84 (T)	1.50 ± 0.84 (C) 1.80 ± 0.52 (T)		1.95 ± 0.46 (C) 2.05 ± 0.57 (T)	NR	10 mL	3000 × 10
Pavani et al. (2021)	3.45 ± 0.34 (C) 4.30 ± 0.73 (T)			2.02 ± 0.64 (C) 2.51 ± 0.91 (T)	NR	6 mL	3000 × 10
Alshoiby et al. (2023)	2.80 ± 0.42 (C) 3.50 ± 1.18 (T)	2.50 ± 0.85 (C) 2.40 ± 0.70 (T)		2.72 ± 1.00 (C) 2.63 ± 0.95 (T)	(Digital Tabletop Centrifuge) Velab, VE‐4000, TX, USA	10 mL	700 × 3
Baghele et al. (2023)	2.76 ± 0.99 (C) 4.10 ± 1.18 (T)	2.23 ± 0.83 (C) 3.57 ± 1.08 (T)		1.67 ± 1.11 (C) 3.38 ± 1.78 (T)	NR	1–3 × 10 mL	3000 × 10
Chaudhary et al. (2023)	4.29 ± 0.97(C) 4.59 ± 1.08 (T)	4.23 ± 1.09 (C) 4.47 ± 1.23 (T)		2.48 ± 0.97(C) 3.83 ± 0.97 (T)	REMI R‐8C (India)	10 mL	2400–2700 × 2
(12) GTR vs. GTR + PRF							
Panda et al. (2016)	3.19 ± 1.33 (C) 3.88 ± 1.15 (T)	3.38 ± 1.45 (C) 4.44 ± 1.50 (T)		0.80 ± 0.28 (C) 2.10 ± 0.64 (T)	Model C‐854/6 (REMI, Mumbai, India)	5 mL	3000 × 10
(13) EMD vs. EMD + PRF							
Aydemir Turkal et al. (2016)	3.88 ± 1.26 (C) 4.00 ± 1.38 (T)	3.29 ± 1.30 (C) 3.42 ± 1.28 (T)		1.21 ± 1.24 (C) 1.13 ± 0.83 (T)	Mikro 22 R (Hettich, Tuttlingen, Germany)	10 mL	400 g × 10
(14) BG + BM vs. BG + BM + PRF							
Liu et al. (2021)	2.40 ± 1.05 (C) 2.70 ± 0.85 (T)	2.70 ± 1.25 (C) 1.80 ± 0.95 (T)			Anke (TDL80‐2B, Shanghai, China)	10 mL	700 × 3
Group V: Addition of biomaterial/biomolecule to PRF							
(15) PRF vs. PRF + BG							
Lekovic et al. (2012)	3.35 ± 0.68 (C) 4.47 ± 0.78 (T)	2.24 ± 0.73 (C) 3.82 ± 0.78 (T)	2.21 ± 0.68 (C) 4.06 ± 0.87 (T)		Labo‐fuge 300; Heraus GmbH, Hanau, Germany	10 mL	1000 g × 10
Chandradas et al. (2016)	3.82 ± 0.75 (C) 4.25 ± 1.48 (T)	3.27 ± 0.65 (C) 3.92 ± 0.90 (T)		2.30 ± 0.83 (C) 3.32 ± 0.72 (T)	R‐4C (REMI, Mumbai, India)	10 mL	3000 × 12
Agarwal et al. (2017)	3.50 ± 1.95 (C) 4.50 ± 2.83 (T)	2.00 ± 2.25 (C) 3.00 ± 3.28 (T)		1.50 ± 2.14 (C) 2.50 ± 2.14 (T)	NR	NR	NR
Pradeep et al. (2017)	3.90 ± 1.09 (C) 4.27 ± 0.98 (T)	3.03 ± 1.16 (C) 3.67 ± 1.03 (T)		3.20 ± 0.89 (C) 3.87 ± 1.33 (T)	R‐4C (REMI, Mumbai, India)	10 mL	3000 × 10
Bahammam and Attia (2021)	2.7 ± 0.89 (C) 3.0 ± 0.94 (T)	1.17 ± 1.21 (C) 2.1 ± 1.04 (T)		2.2 ± 0.09 (C) 2.31 ± 1.50 (T)	NR	5 mL	3000 × 10
Thetay et al. (2021)	3.90 ± 0.99 (C) 4.10 ± 0.85 (T)	4.10 ± 1.60 (C) 4.50 ± 0.97 (T)		3.70 ± 1.16 (C) 4.80 ± 1.03 (T)	R‐4C (REMI, Mumbai, India)	10 mL	3000 × 10
(16) PRF vs. PRF + AA							
Elbehwashy et al. (2021)	4.10 ± 0.61 (C) 3.45 ± 1.36 (T)	3.90 ± 1.15 (C) 4.25 ± 1.27 (T)		1.63 ± 0.46 (C) 2.29 ± 0.61 (T)	NR	10 mL	3000 × 10
(17) PRF vs. PRF + Metformin							
Pradeep et al. (2015)	4.00 ± 0.18 (C) 4.90 ± 0.30 (T)	4.03 ± 0.18 (C) 4.90 ± 0.30 (T)		2.53 ± 0.30 (C) 2.77 ± 0.30 (T)	R‐4C (REMI, Mumbai, India)	10 mL	3000 × 10
(18) PRF vs. PRF + Bisphosphonates							
Kanoriya et al. (2016)	3.70 ± 0.91 (C) 4.53 ± 0.81 (T)	4.20 ± 0.66 (C) 5.16 ± 0.46 (T)		2.42 ± 0.21 (C) 2.84 ± 0.26 (T)	R‐4C (REMI, Mumbai, India)	10 mL	3000 × 10
(19) PRF vs. PRF + Statins							
Martande et al. (2016)	3.76 ± 1.12 (C) 4.06 ± 1.22 (T)	3.40 ± 1.13 (C) 3.66 ± 1.42 (T)		2.46 ± 0.33 (C) 2.58 ± 0.36 (T)	R‐4C (REMI, Mumbai, India)	5 mL	3000 × 12 to 14
Pradeep et al. (2016)	4.03 ± 0.18 (C) 4.90 ± 0.31 (T)	3.30 ± 0.65 (C) 3.93 ± 0.78 (T)		3.17 ± 0.65 (C) 3.63 ± 0.67 (T)	R‐4C (REMI, Mumbai, India)	10 mL	3000 × 10
(20) BG + PRF vs. BG + PRF + Statins							
Gautam et al. (2022)	3.38 ± 0.51 (C) 4.46 ± 0.78 (T)	2.77 ± 0.59 (C) 4.00 ± 0.71 (T)		2.90 ± 0.17 (C) 4.25 ± 0.29 (T)	NR	10 mL	3000 × 10
(21) PRF vs. PRF + LLLT							
Thalaimalai et al. (2020)	2.94 ± 0.90 (C) 3.60 ± 1.00 (T)	2.26 ± 1.37 (C) 3.27 ± 1.51 (T)		1.26 ± 1.48 (C) 2.44 ± 1.52 (T)	NR	10 mL	3000 × 10

Abbreviations: ♀, female; ♂, male; AA, ascorbic acid; ABBM, anorganic bovine bone mineral; ABG, autogenous bone graft; ALN, alendronate; ATV, atorvastatin; BCP, bicalcium phosphate; BF, bone fill; BG, bone graft; BioG, BioGlass, bioactive glass; BM, barrier membrane; BM, barrier membrane; BPBM, bovine porous bovine mineral; C, control group; CAL, clinical attachment level; CPS, calcium phosphosilicate; DBM, demineralized bone matrix; DFDBA, demineralized freeze‐dried bone allograft; EMD, enamel matrix derivative; GAgp, Generalized aggressive periodontitis; GTR, guided tissue regeneration; HA, hydroxyapatite; IBDs, infrabony defects; I‐PRF, injectable PRF; LLL, low‐level laser therapy; L‐PRF, Leukocyte‐PRF; MF, metformin; min, minute; M‐MIST, modified‐minimally invasive surgical technique; NR, not reported; OFD, open flap debridement; PD, probing depth; PRF, platelet‐rich fibrin; PRP, platelet‐rich plasma; RCT, randomized clinical trial; RPM, rotation per minute; RSV, rosuvastatin; SPPF, simplified papilla preservation flap; T, test group; T‐PRF, titanium‐PRF; XB, Xeno, xenogeneic bone; β‐TCP, beta‐tricalcium phosphate.

### 3.2 Study characteristics

The included studies analyzed 1025 research participants. In addition to OFD alone, the effect of PRF was compared with other groups of biomaterials (autograft, allograft, xenograft, alloplast, barrier membrane, EMD, metformin, bisphosphonates, and statins). The mean follow‐up period of the studies was 8.44 ± 2.04 months. The data extracted from each included study are presented in Table 1.

### 3.3 Interventions and comparisons

A total of 21 categories were divided into five groups as follows:

**Group I: OFD versus OFD/PRF**

1. OFD versus OFD/PRF
2. OFD versus OFD/T‐PRF

**Group II: Comparative protocols of PRF**

3 OFD/PRF versus OFD/T‐PRF

**Group III: Comparative studies to PRF**

4 OFD/PRP versus OFD/PRF
5 OFD/BG/PRGF versus OFD/BG/PRF
6 OFD/EMD versus OFD/PRF
7 OFD/BG/EMD versus OFD/BG/PRF
8 OFD/BM versus OFD/PRF
9 OFD/BG/BM versus OFD/BG/PRF
10 OFD/BG versus OFD/PRF

**Group IV: Addition of PRF to a treatment modality**

11 OFD/BG versus OFD/BG/PRF
12 OFD/GTR versus OFD/GTR/PRF
13 OFD/EMD versus OFD/EMD/PRF
14 OFD/BG/BM versus OFD/BG/BM/PRF

**Group V: Addition of biomaterials/biomolecules to PRF**

15 OFD/PRF versus OFD/PRF/BG
16 OFD/PRF versus OFD/PRF/Antibiotic (AA)
17 OFD/PRF versus OFD/PRF/Metformin
18 OFD/PRF versus OFD/PRF/Bisphosphonate
19 OFD/PRF versus OFD/PRF/Statins
20 OFD/BG/PRF versus OFD/BG/PRF/Statins
21 OFD/PRF versus OFD/PRF/LLLT (low‐level laser therapy)

#### 3.3.1 Group I: OFD versus OFD/PRF

For Group I, two comparative subgroups were analyzed (OFD vs. PRF [21 trials] and OFD vs. T‐PRF [one trial Ustaoglu 2020]).

##### Probing pocket depth

**FIGURE 2:**
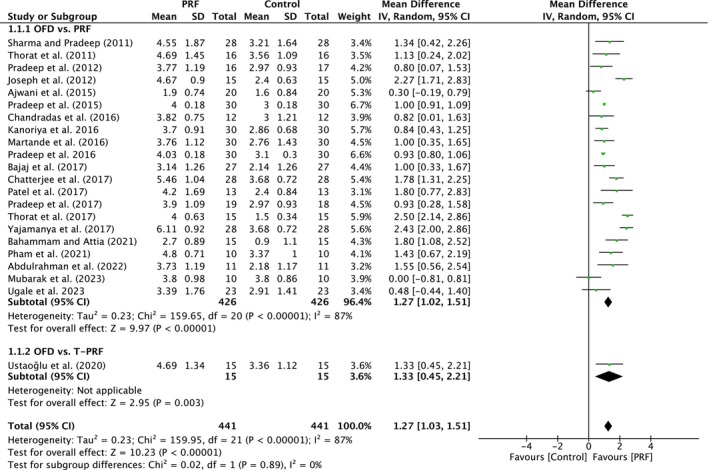
Forest plot for the event reduction in “probing pocket depth” (PPD) (reported in mm) for intrabony defects for Group I: OFD versus OFD/PRF.

A total of 22 studies were analyzed. A high heterogeneity was observed between the studies (*I* ^2^ = 87%; *p* < 0.00001). The overall effect was 1.27 mm (95% CI: 1.03 to 1.51; *p* < 0.00001) with no significant difference between the subgroups (*p* = 0.89). The subgroups demonstrated a significant effect (*p* < 0.00001; *p* = 0.003) when compared to OFD alone, with MD of 1.27 mm (95% CI: 1.02 to 1.51) and MD of 1.33 mm (95% CI: 0.45 to 2.21) (Figure 2).

##### Clinical attachment level

**FIGURE 3:**
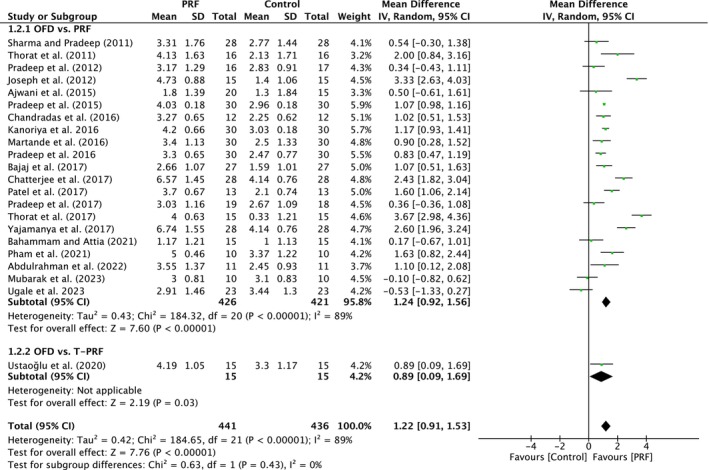
Forest plot for the event “clinical attachment level” (CAL) (reported in mm) for intrabony defects for Group I: OFD versus OFD/PRF.

A total of 22 studies were analyzed. A high heterogeneity was observed between the studies (*I* ^2^ = 89%; *p* < 0.00001). The overall effect was 1.22 mm (95% CI: 0.91 to 1.53; *p* < 0.00001) with no significant difference between the subgroups (*p* = 0.43). The subgroups demonstrated a significant effect (*p* < 0.00001; *p* = 0.03) when compared to OFD alone, with MD of 1.24 mm (95% CI: 0.92 to 1.56) and MD of 0.89 mm (95% CI: 0.09 to 1.69) (Figure 3).

##### Radiographic bone fill

**FIGURE 4:**
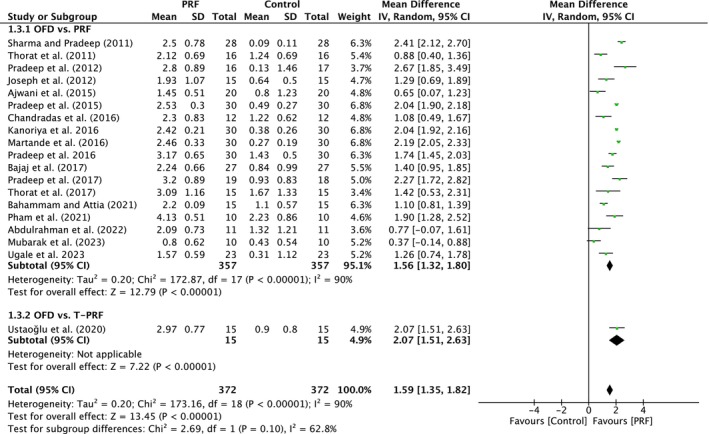
Forest plot for the event “radiographic bone fill” (RBF) (reported in mm) for intrabony defects for Group I: OFD versus OFD/PRF.

A total of 19 studies were analyzed. A high heterogeneity was observed between the studies (*I* ^2^ = 90%; *p* < 0.00001). The overall effect was 1.59 mm (95% CI: 1.35 to 1.82; *p* < 0.00001) with no significant difference between the subgroups (*p* = 0.10). The subgroups demonstrated a significant effect (*p* < 0.00001; *p* < 0.00001) when compared to OFD alone, with MD of 1.56 mm (95% CI: 1.32 to 1.80) and MD of 2.07 mm (95% CI: 1.51 to 2.63) (Figure 4).

##### Bone sounding/bone fill

There were no studies providing data for this outcome.

#### 3.3.2 Group II: Comparative protocols of PRF

For Group II, comparative PRF protocols were analyzed (PRF vs. T‐PRF [two trials]).

##### Probing pocket depth

**FIGURE 5:**
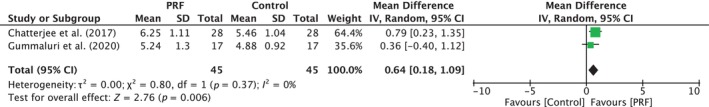
Forest plot for the event reduction in “probing pocket depth” (PPD) (reported in mm) for intrabony defects for Group II: Comparative Protocols of PRF.

A total of two studies were analyzed. No heterogeneity was observed between the studies (*I* ^2^ = 0%; *p* = 0.37). There was a significant difference between the groups analyzed in favor of PRF (*p* = 0.006), with MD of 0.64 mm (95% CI: 0.18 to 1.09) (Figure 5).

##### Clinical attachment level

**FIGURE 6:**
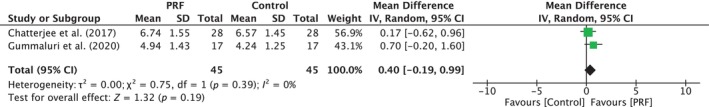
Forest plot for the event “clinical attachment level” (CAL) (reported in mm) for intrabony defects for Group II: Comparative Protocols of PRF.

A total of two studies were analyzed. No heterogeneity was observed between the studies (*I* ^2^ = 0%; *p* = 0.39). There was no significant difference between the groups analyzed (*p* = 0.19), with MD of 0.40 mm (95% CI: −0.19 to 0.99) (Figure 6).

##### Radiographic bone fill

Only one study provided data for this outcome.^64^ A significant mean bone fill in both groups (*p* < 0.001) was found, but more advantageously outcomes were found with the T‐PRF treatment (*p* = 0.02).

##### Bone sounding/bone fill

There were no studies providing data for this outcome.

#### 3.3.3 Group III: Comparative studies to PRF

For Group III, seven comparative subgroups including 15 studies were analyzed (PRP vs. PRF [one trial]; BG/PRGF vs. BG/PRF [one trial]; EMD vs. PRF [two trials]; BG/EMD vs. BG/PRF [one trial]; BM vs. PRF [two trials]; BG/BM vs. BM/PRF [two trials]; BG vs. PRF [six trials]). All treatments were performed in combination with OFD.

##### Probing pocket depth

**FIGURE 7:**
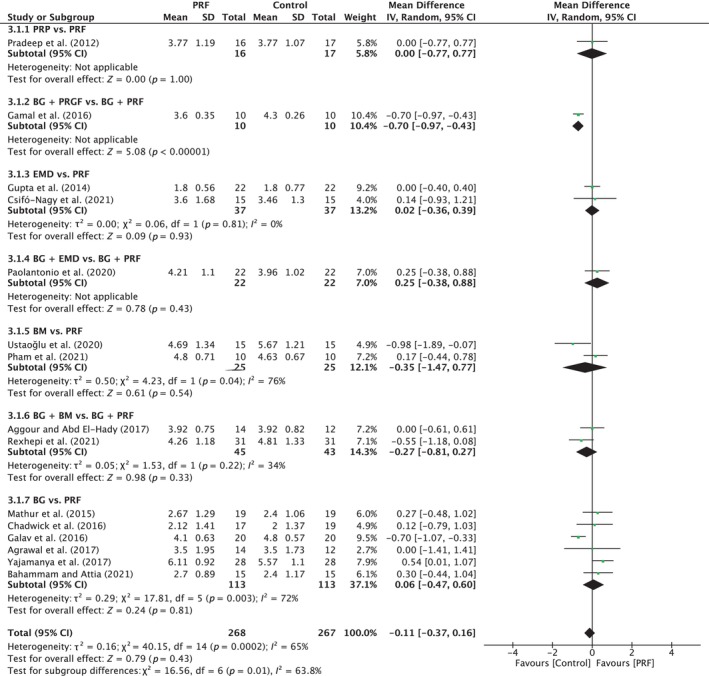
Forest plot for the event reduction in “probing pocket depth” (PPD) (reported in mm) for intrabony defects for Group III: Comparative studies to PRF.

A total of 15 studies were analyzed. A high heterogeneity was observed between the studies (*I* ^2^ = 65%; *p* = 0.0002). The overall effect was −0.11 mm (95% CI: −0.37 to 0.16; *p* = 0.43) in favor of control with a significant difference between the subgroups (*p* = 0.01) (Figure 7).

##### Clinical attachment level

**FIGURE 8:**
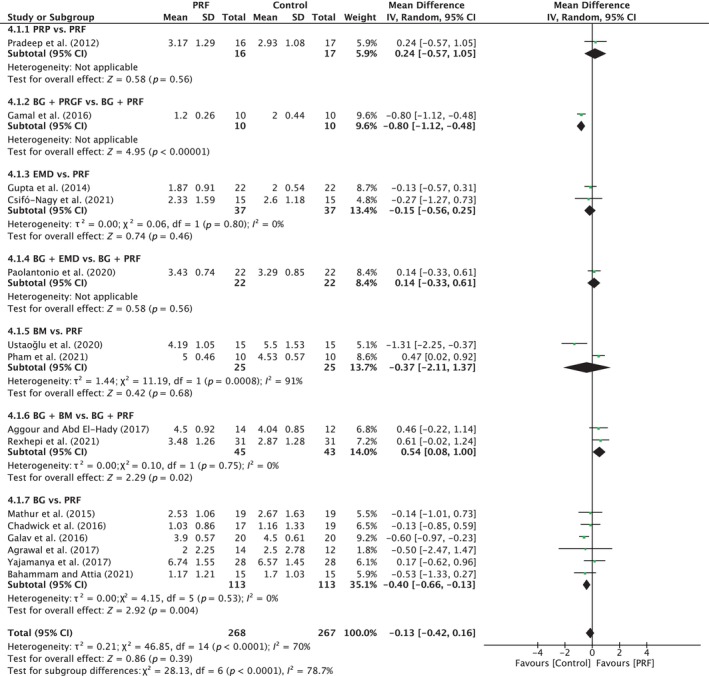
Forest plot for the event “clinical attachment level” (CAL) (reported in mm) for intrabony defects for Group III: Comparative studies to PRF.

A total of 15 studies were analyzed. A high heterogeneity was observed between the studies (*I* ^2^ = 70%; *p* < 0.0001). The overall effect was −0.13 mm (95% CI: −0.42 to 0.16; *p* = 0.39) in favor of control with a significant difference between the subgroups (*p* < 0.0001). The only significant differences were found for BG/BM versus BG/PRF (two studies) in favor of PRF (*p* = 0.02), with MD of 0.54 mm (95% CI: 0.08 to 1.00) and for BG versus PRF (six studies) in favor of BG (*p* = 0.004) with MD of −0.40 mm (95% CI: −0.66 to −0.13) (Figure 8).

##### Radiographic bone fill

**FIGURE 9:**
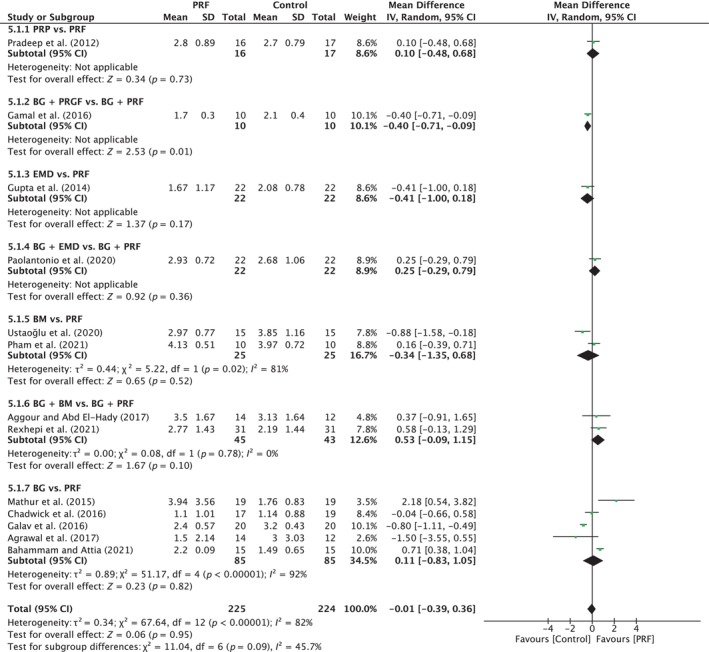
Forest plot for the event “radiographic bone fill” (RBF) (reported in mm) for intrabony defects for Group III: Comparative studies to PRF.

A total of 13 studies were analyzed. A high heterogeneity was observed between the studies (*I* ^2^ = 82%; *p* < 0.00001). The overall effect was −0.01 mm (95% CI: −0.39 to 0.36; *p* = 0.95) in favor of control with no significant difference between the subgroups (*p* = 0.09) (Figure 9).

##### Bone sounding/bone fill

There were four trials providing data for bone fill as assessed by bone sounding or reentry (BS/BF) (Table 1).

#### 3.3.4 Group IV: Addition of PRF to a treatment modality

Four subgroups including 20 trials were analyzed for Group IV BG versus BG/PRF (17 trials); GRT versus GTR/PRF (one trial); EMD versus EMD/PRF (one trial); and BG/BM versus BG/BM/PRF (one trial). All treatments performed in combination with OFD.

##### Probing pocket depth

**FIGURE 10:**
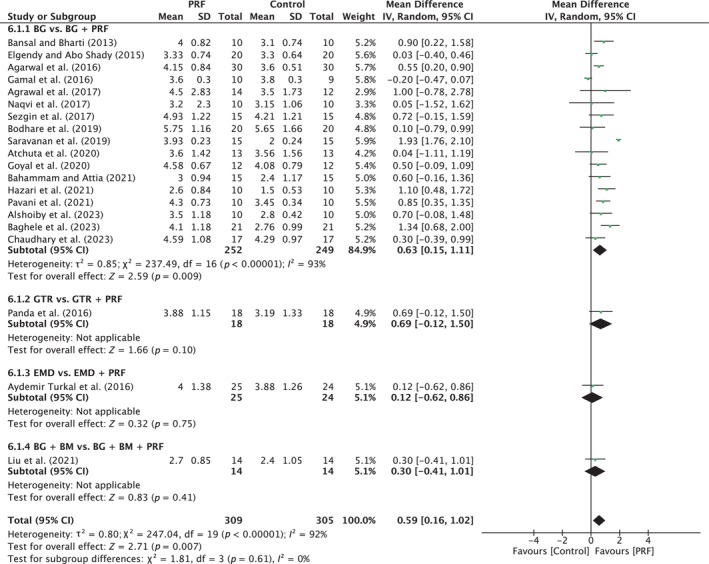
Forest plot for the event reduction in “probing pocket depth” (PPD) (reported in mm) for intrabony defects for Group IV: Addition of PRF to a treatment modality.

A total of 20 studies were analyzed. A high heterogeneity was observed between the studies (*I* ^2^ = 92%; *p* < 0.00001). The overall effect was 0.59 mm (95% CI: 0.16 to 1.02; *p* = 0.007) in favor of PRF with no significant difference between the subgroups (*p* = 0.09). Only one subgroup (BG vs. BG/PRF) showed a significant difference in favor of the PRF group, with MD of 0.63 mm (95% CI: 0.15 to 1.11; *p* = 0.009), with a high degree of heterogeneity (*I* ^2^ = 93%; *p* < 0.00001) (Figure 10).

##### Clinical attachment level

**FIGURE 11:**
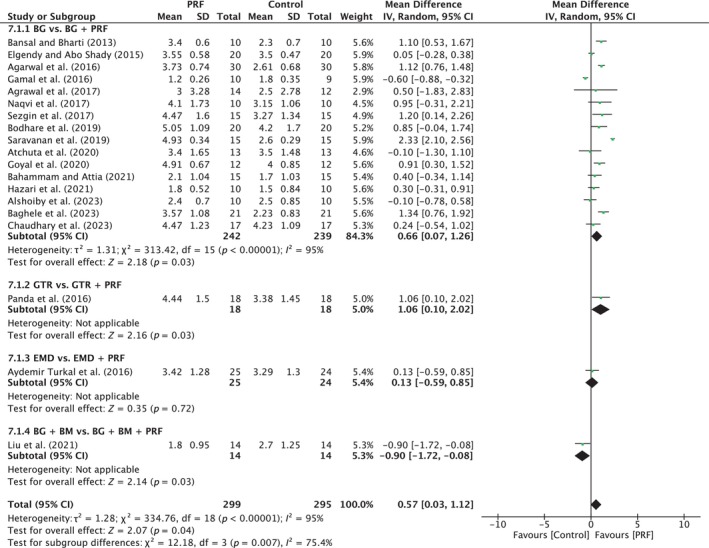
Forest plot for the event “clinical attachment level” (CAL) (reported in mm) for intrabony defects for Group IV: Addition of PRF to a treatment modality.

A total of 19 studies were analyzed. A high heterogeneity was observed between the studies (*I* ^2^ = 95%; *p* < 0.00001). The overall effect was 0.57 mm (95% CI: 0.03 to 1.12; *p* = 0.04) in favor of the PRF group with a significant difference between subgroups (*p* = 0.007). BG versus BG/PRF showed a significant difference in favor of the PRF group, with MD of 0.66 mm (95% CI: 0.07 to 1.26; *p* = 0.03) (Figure 11).

##### Radiographic bone fill

**FIGURE 12:**
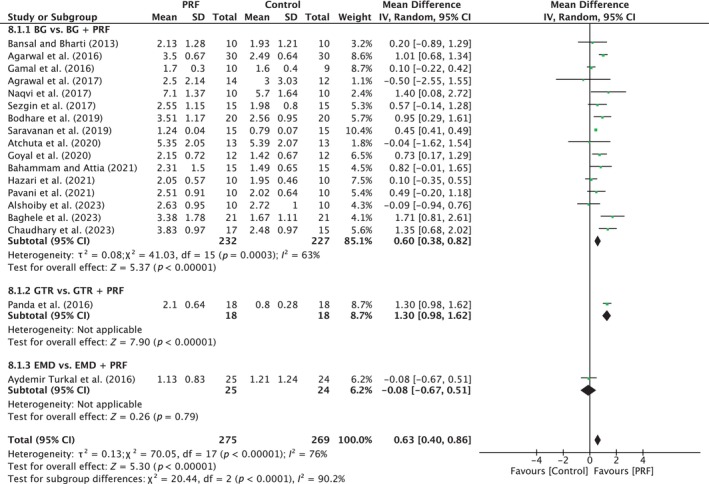
Forest plot for the event “radiographic bone fill” (RBF) (reported in mm) for intrabony defects for Group IV: Addition of PRF to a treatment modality.

A total of 18 studies were analyzed. A high heterogeneity was observed between the studies (*I* ^2^ = 76%; *p* < 0.00001). The overall effect was 0.63 mm (95% CI: 0.40 to 0.86; *p* < 0.00001) in favor of the test group with a significant difference between the subgroups (*p* < 0.0001). BG versus BG/PRF showed a significant difference in favor of the test group with MD of 0.60 mm (95% CI: 0.38 to 0.82; *p* < 0.00001; 16 studies) (Figure 12).

##### Bone sounding/bone fill

Only one trial provided data for BS/BF.^85^ A significant mean bone fill within both groups (*p* < 0.001) was found, but between groups, no difference was noticed (*p* = 0.653).

#### 3.3.5 Group V: Addition of biomaterials/biomolecules to PRF

For Group V, seven comparative subgroups including 13 studies were analyzed in 13 trials (PRF vs. PRF/BG [six studies]; PRF vs. PRF/Antibiotic [one study]; PRF vs. PRF/Metformin [one study]; PRF vs. PRF/Bisphosphonate [one study]; PRF vs. PRF/Statins [two studies]; PRF/BG vs. PRF/BG/Statins [one study]; PRF vs. PRF/LLLT [one study]).

##### Probing pocket depth

**FIGURE 13:**
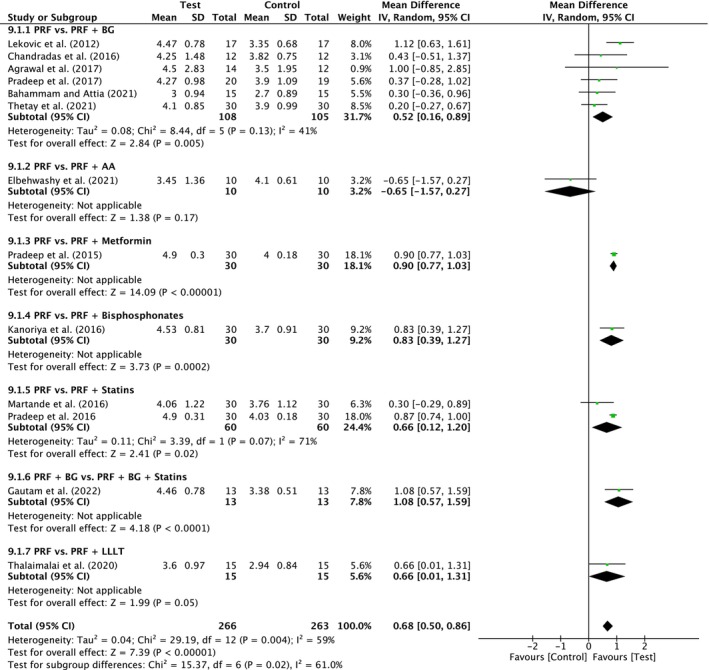
Forest plot for the event reduction in “probing pocket depth” (PPD) (reported in mm) for intrabony defects for Group V: Addition of Biomaterials/Biomolecules to PRF.

A total of 13 studies were analyzed. A high heterogeneity was observed between the studies (*I* ^2^ = 59%; *p* = 0.004). The overall effect was 0.68 mm (95% CI: 0.50 to 0.86; *p* < 0.00001) in favor of test with a significant difference between the subgroups (*p* = 0.02). There was a significant difference for PRF versus PRF/BG (six studies) in favor of test with MD of 0.52 mm (95% CI: 0.16 to 0.89; *p* = 0.005) and for PRF/BG versus PRF/BG/Statins (two studies) in favor of test with MD of 0.66 mm (95% CI: 0.12 to 1.20; *p* = 0.02) (Figure 13). Other subgroups showed also significant differences, but for each comparison, only one trial was included, respectively. Only PRF versus PRF/Antibiotic showed no significant advantage for the additional treatment.

##### Clinical attachment level

**FIGURE 14:**
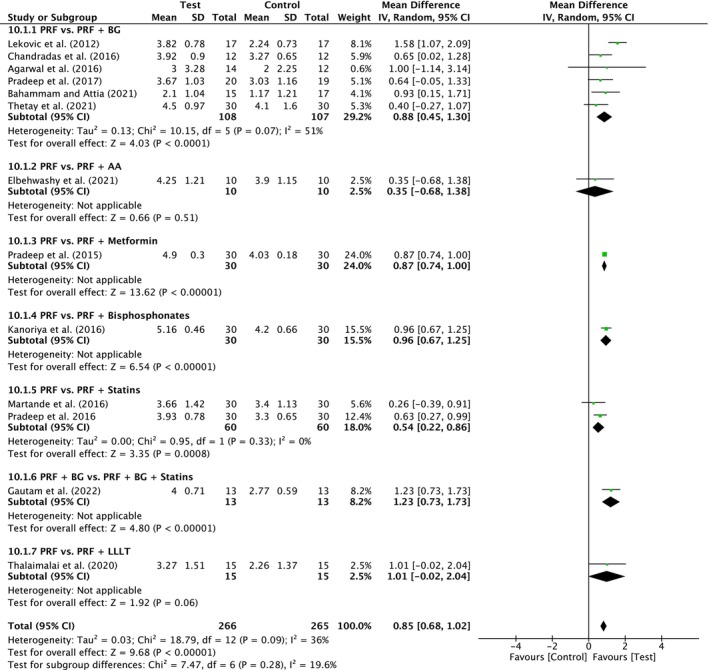
Forest plot for the event “clinical attachment level” (CAL) (reported in mm) for intrabony defects for Group V: Addition of Biomaterials/Biomolecules to PRF.

A total of 13 studies were analyzed. A moderate heterogeneity was observed between the studies (*I* ^2^ = 36%; *p* = 0.09). The overall effect was 0.85 mm (95% CI: 0.68 to 1.02; *p* < 0.00001) in favor of test treatments with no significant difference between the subgroups (*p* = 0.28). There was only a significant difference for PRF versus PRF/BG (six studies) in favor of test with MD of 0.88 mm (95% CI: 0.45 to 1.30; *p* < 0.0001) and for PRF/BG versus PRF/BG/Statins (two studies) in favor of test with MD of 0.54 mm (95% CI: 0.22 to 0.86; *p* = 0.0008) (Figure 14). Other subgroups showed also significant differences, but for each comparison, only one trial was included, respectively. Only PRF versus PRF/Antibiotic and PRF versus PRF/LLLT showed no significant advantage for the additional treatment.

##### Radiographic bone fill

**FIGURE 15:**
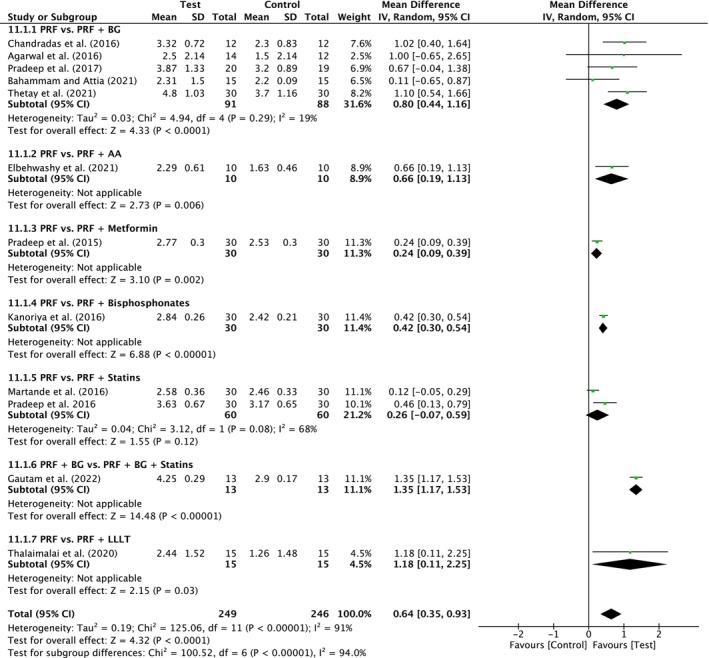
Forest plot for the event “radiographic bone fill” (RBF) (reported in mm) for intrabony defects for Group V: Addition of Biomaterials/Biomolecules to PRF.

A total of 12 studies were analyzed. A high heterogeneity was observed between the studies (*I* ^2^ = 93%; *p* < 0.00001) with a significant difference between the subgroups (*p* < 0.00001). There was a significant difference for PRF versus PRF/BG (five studies) in favor of test with MD of 0.80 mm (95% CI 0.44 to 1.16; *p* < 0.0001). All other comparisons were only with one study each, besides one comparison of PRF versus PRF/Statins where no significant higher bone fill on radiograms with the combined treatment was found (*p* = 0.12; two trials) (Figure 15).

##### Bone sounding/bone fill

Only one trial provided data for BS/BF.^92^ A significantly greater bone fill was found with the test treatment (*p* < 0.001).

### 3.4 Overall risk of bias

Fourteen studies were assessed with low risk of bias in all five domains, four trials were at high risk of bias in at least one domain,^57^, ^71^, ^81^, ^92^ and the remaining 37 studies of the included 55 studies were with some concerns in at least one domain. For 24 studies of the 37 studies with uncertain risk of bias (some concerns), the decision was related to the missing information of allocation concealment, for example, sealed envelopes only. If this had been clearly stated in the description of the randomization process in the publications, those 24 studies would also have been with an overall low risk of bias (Appendix S1).

### 3.5 Randomization process

Forty‐nine trials described clearly the method of randomization. Twenty‐one used a computer‐assisted method, in 28 trials, the method of randomization was a coin toss or lottery method. Fifteen studies used sealed envelopes in combination with an additional randomization process and were deemed with low risk in this domain. In six trials, the method of randomization was uncertain.^75^, ^76^, ^81^, ^85^, ^95^, ^96^

### 3.6 Deviations from the intended interventions

We did not score performance bias of the operator as it was impossible to blind the therapist to the surgical intervention. Fifty trials were with low risk of bias for this domain. Five studies gave neither information of power calculations nor of the dropout rate and were deemed with unclear risk of bias (some concerns).^66^, ^69^, ^76^, ^85^, ^96^

### 3.7 Missing outcome data

All trials were with low risk of bias for this domain. However, there was some lack of information about dropout rate in some studies. Still, in those cases it was reasonable on basis of tables and figures to assume, that all missingness probably did not depend on its true value.

### 3.8 Measurement of the outcome

Forty‐one studies were with low risk of bias, the remaining 11 studies were with some concerns due to missing information of outcome assessor being blinded. Three trials were with high risk of bias, due to written information that assessors were not blinded,^57^ unorthodox, unexplained measurement methods^71^ or lack of important information.^81^

### 3.9 Selection of the reported result

Fifty‐four trials were found with low risk of bias for this domain, one study with unclear risk of bias due to insufficient information about measuring methods.^71^ The ROB 2 analysis is shown in the Appendix S1.

## 4 DISCUSSION

The present SR and meta‐analysis have investigated the use of PRF for reconstructive surgery in intrabony defects as evaluated in RCTs comparing it to other treatment modalities. The aim was to more specifically address the use and recommendations for PRF for the treatment of periodontal two‐ and three‐walled intrabony defects. Overall, the majority of the included studies compared the use of OFD alone versus OFD/PRF, OFD/BG versus OFD/PRF, and OFD/BG versus OFD/BG/PRF (Table 1). Furthermore, additional studies were gathered comparing OFD/EMD versus OFD/PRF, OFD/BM versus OFD/PRF, and OFD/PRF versus OFD/PRF/Biomolecules. Below, we highlight and discuss the summary of evidence from the current categories and further discuss the strengths and limitations of each comparative analysis.

### 4.1 Group I: OFD alone versus OFD/PRF

In total, 23 studies, five with low, 16 with unclear, and one with high risk of bias, evaluated the use of PRF (22 studies) or T‐PRF (one study) as an adjunct to OFD when compared to OFD alone (Table 1). In summary, 20 of the 23 studies demonstrated statistically significant clinical improvements in mean PPD reduction (Figure 2), 16 studies statistically significant improvements in mean CAL gain (Figure 3), and 18 studies statistically significant improvements in bone fill (Figure 4). It was observed that on average, the results from 23 RCTs demonstrated a statistically significant additional PPD reduction of ~1.3, ~1.2 mm CAL gain, and ~1.5 radiographic bone gain when PRF was applied to intrabony defects following OFD.

These findings, although based on a much higher number of studies and participants, are in agreement with a recently published overview of SRs^97^ and exceed the results reported by the AAP best evidence SR and network meta‐analysis.^25^ Notably, the present results are also in concert with the overall MDs following various pooled regenerative procedures compared with access flap reported by the SR with meta‐analysis^8^ that was prepared for the EFP S3‐Level CPG on the treatment of periodontitis.^9^ Thus, to date there is robust evidence that the use of PRF as autogenous biologics during access flap surgery can yield clinical outcomes similar to those of established regenerative procedures, without the expenses for nonautogeneous commercial biomaterials.

### 4.2 Group II: L‐PRF versus Titanium‐PRF

Only two studies, both with unclear risk of bias, compared in an RCT the effects of different protocols to produce PRF on intrabony defect regeneration (Figures 5 and 6). The meta‐analysis demonstrated a small significant improvement of 0.64 mm for PPD using T‐PRF when compared to PRF (Figure 5) while no advantage was found for CAL (Figure 6). Another SR^24^ included three studies comparing PRF and T‐PRF,^53^, ^64^, ^98^ however did not perform a meta‐analysis. A significantly greater defect fill in favor of T‐PRF was reported by Gummaluri et al.,^64^ whereas no differences for any of the parameters were seen in the other two studies. One important discussion point is that in the study by Gummaluri et al.^64^ the authors utilized both: differences in PRF tubes (titanium tubes vs. standard tubes) and a different centrifugation protocol. Therefore, the data should interpret with caution as the authors had two variable parameters in their study (both centrifugation protocol and tube type) and it is difficult to draw conclusions on which of those two parameters was more relevant. Future studies comparing the impact of PRF protocols are desirable as many improvements in growth factor release and concentration of cells have been reported more recently as discussed below.^99^

### 4.3 Group III: Comparative studies to PRF

A total of seven different categories compared either OFD/PRP, OFD/EMD, OFD/BM, or OFD/BG to OFD/PRF, as well as OFD/BG/PRGF to OFD/BG/PRF, OFD/BG/EMD to OFD/BG/PRF, and OFD/BG/BM to OFD/BG/PRF. A total of 15 studies, four with low, nine with unclear, and two with high risk of bias, were included in the comparisons.

Significant differences were calculated for CAL gain (Figure 8): (1) higher mean CAL gain of 0.54 mm in the OFD/BG/PRF group (two studies) versus OFD/BG/BM and (2) higher mean CAL gain of 0.40 mm in the OFD/BG group (six studies) versus OFD/PRF. While it is known that PRF cannot hold space/volume as effectively as a BG, it is noteworthy that five of the six studies depicted no significant differences in CAL gain between the two groups. Noteworthy, only one study compared OFD/PRP to OFD/PRF with more research needed investigating the benefit of various formulations of APCs on intrabony defect regeneration.

### 4.4 Group IV: Addition of PRF to a treatment modality

A number of studies have compared the additional use of PRF, most commonly when added to a bone grafting material (Table 1). The included 20 studies—6 with low, 13 with unclear, and 1 with high risk of bias—showed significant mean improvements of about 0.6 mm in PPD reduction, as well as in CAL and RBF gain, when PRF was added (Figures 10, 11, 12). Therefore, the data indicate that the addition of PRF may lead to enhanced clinical outcomes compared with those obtained with BG alone in the regneration of intrabony defects. These findings are in line with the conclusions of the recent AAP best evidence SR stating that combination therapies involving bone grafts + biologics such as PRF demonstrate to be superior than monotherapies.^25^

Potential reasons for these findings are likely multi‐factorial. Many bone grafting materials, such as xenografts and the majority of synthetic materials, have no incorporation of extracellular matrix components or growth factors. Therefore, one hypothesized reason for the additional benefit of including PRF to a BG could be its newly incorporation of regenerative cells and growth factors that contribute to the regenerative process. Previous in vitro research investigating PRF has demonstrated its ability to improve PDL cell migration, proliferation, and wound closure rates.^100^ Furthermore, PRF also contains supra‐physiological concentrations of defense‐fighting leukocytes. Since periodontal pockets harbor a number of periodontal pathogens, it is possible that leukocytes may aid in the defense against potential bacterial contamination/invasion. Lastly, basic science studies have now demonstrated that PRF promotes an anti‐inflammatory environment.^101^, ^102^ Recent research has shown that PRF has the ability to favor M2 macrophage polarization and also decreases tissue inflammation.^101^, ^102^ Furthermore, a recent SR by our group demonstrated that PRF also possesses anti‐bacterial/antimicrobial activity, thereby favoring potential wound healing of periodontal pockets.^103^ Taken together, each of the above‐mentioned parameters is thought to at least in part contribute toward periodontal regeneration when PRF is utilized in combination with a BG.

No clinical or radiological improvements were observed when PRF was added or mixed with EMD. This likely was due to both acting via similar mechanisms in terms of growth factors' improvements in cell recruitment and/or activity using biologic agents.

### 4.5 Group V: Addition of biomaterials/biomolecules to PRF

An additional 13 studies (four with low, eight with unclear, and one with high risk of bias) evaluated the addition of a biomaterial/biomolecule to PRF (Table 1). First, the addition of a bone graft to PRF (PRF vs. PRF + BG) led to improvements in PPD (0.52 mm, six studies, Figure 13), CAL (0.88 mm, six studies, Figure 14), and bone fill (0.80 mm, five studies, Figure 15) when compared to PRF alone. Future investigating on the presented clinical parameters and walls present/size of the defects may better address when a bone grafting material should be combined with PRF versus PRF alone.

Four studies have investigated PRF in combination with either (1) metformin,^47^ (2) bisphosphonates,^49^ or (3) statins.^50^, ^51^, ^95^ There was a statistically significant advantage in PPD reduction, CAL gain, and RBF for the combined use of PRF with each of the above modalities when compared to utilizing PRF alone, except for PPD reduction with the combination PRF/metformin (Figures 13, 14, 15). Though few studies have thus far characterized their potential benefit, these relatively novel findings support the more recent trends favoring more “personalized” medicine as regenerative strategies. Thus, future research investigating specific patient populations (e.g. osteoporotic women) may potentially and more specifically target the local use of additional biomolecules (such as bisphosphonates) favoring more specific bioactivity (anti‐resorptive properties) favoring a more personalized treatment protocol. Since PRF may be utilized as a three‐dimensional matrix with long‐term delivery of small biomolecules, PRF may therefore be utilized as a therapeutic drug delivery system as previously reported.^104^ Nevertheless, it remains somewhat unclear the modes of action of certain strategies such as an additional combination of Metformin to PRF. Future research investigating PRF as a potential drug delivery system for various local therapeutic agents/biomolecules with their better understanding may provide further clinical benefit. At present, however, the above trends are simply reported in single RCTs with much further research needed on the topic.

### 4.6 Implications for clinical practice and future direction

**FIGURE 16:**
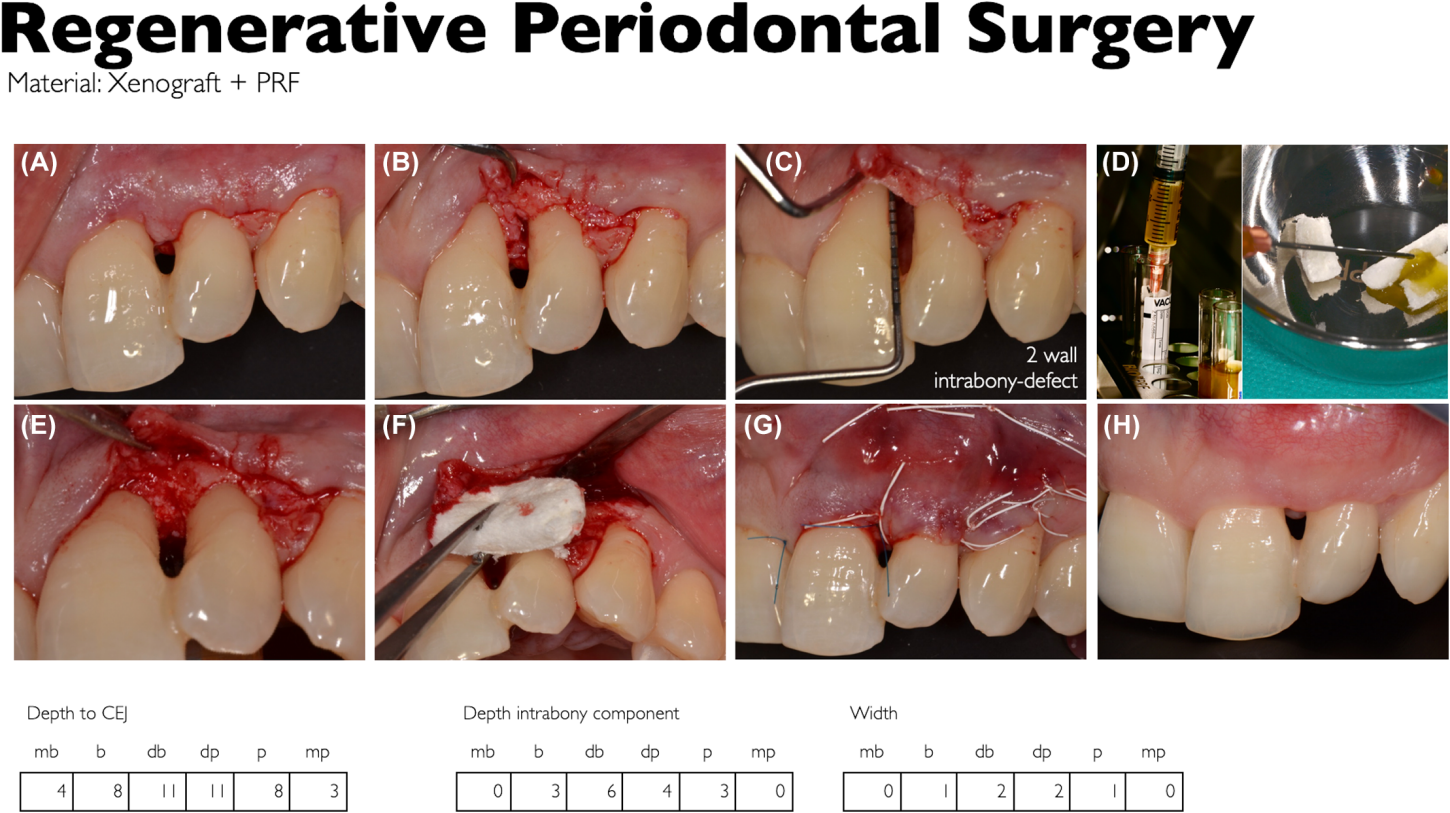
Regenerative periodontal surgery of the upper left lateral incisor (#21) splinted to the neighboring tooth #22. A 2‐wall intrabony defect treated with OFD + Natural Bone Mineral + PRF. A volume stable collagen matrix served as „soft tissue wall“. (A) Intrasulcular incision, extended to the distal for accessibility and tension release, (B) after flap elevation, (C) after removal of granulation tissue, 6‐mm deep intrabony component of the defect, depth to the CEJ: 11 mm, (D) preparation of PRF, (E) defect filled with natural bone mineral mixed with PRF, (F) volume stable collagen matrix served as “soft tissue wall“, (G) clinical view after suturing (sling‐ and single sutures), situation after 4 weeks, healing was uneventful.

**FIGURE 17:**
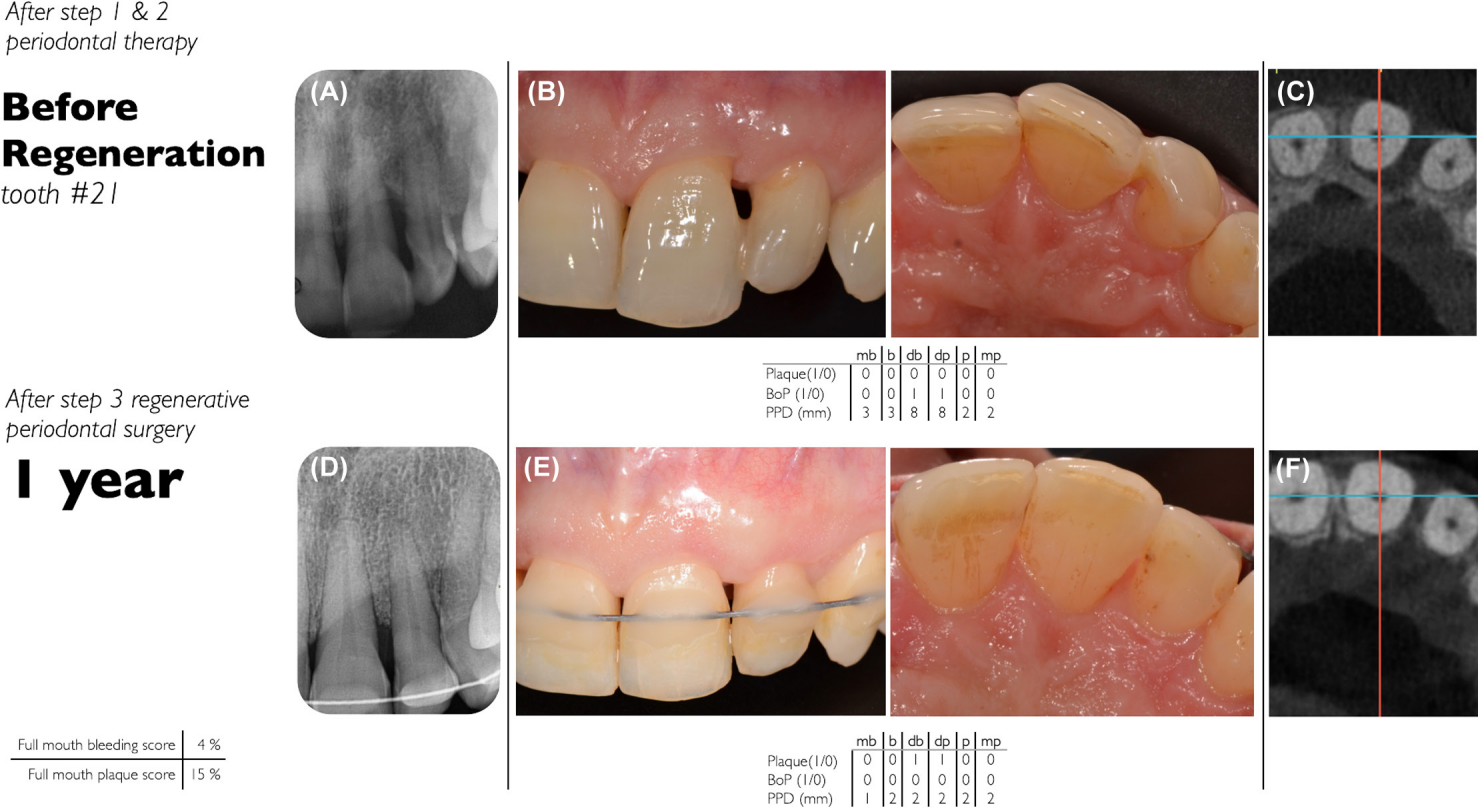
Radiographic and clinical situation of an upper left lateral incisor (#21) in a 45‐year‐old otherwise healthy patient after step 1 and 2 periodontal therapy and 1 year after OFD + Natural Bone Mineral + PRF. (A) Radiographic view with 2‐wall intrabony defect with bone loss ≥60%, (B) clinical attachment loss at the distal aspect 11mm of #21, mobility grade II, (C) CBCT with evidence of severe bone loss around tooth #21 extending to the mesio‐palatal aspect, (D) one‐year follow‐up of lesion with no evidence of radiographic pathologies, (E) clinical view with #21 still splinted with shallow probings, no recession and no bleeding on probing, (F) CBCT view with no more evidence of bone loss.

The S3‐Level EFP CPG^9^ recommended that EMD or GTR in combination with papillary preservation flaps should be considered the treatment of choice for residual pockets with deep (≥3 mm) intrabony defects.^8^ While the fact that blood clot seems to be a very important aspect for periodontal regeneration, it must first be noted that there remains a great need for human histology with lots of effort to deliver studies that compare standard periodontal regenerative procedures with PRF. For the future, inclusion of PRF in an updated version of the CPG for the treatment of periodontitis evidence by human histology is a requirement. Furthermore, RCTs of at least 12 months duration are mandatory. Despite the fact that PRF is only beginning to be more commonly utilized in routine clinical practice for the treatment of intrabony defects (Figures 16 and 17), it is interesting to note that 55 RCTs have thus far evaluated its potential for periodontal regeneration/repair. However, only eight trials had a follow‐up of at least 12 months,^44^, ^45^, ^54^, ^59^, ^68^, ^70^, ^77^, ^91^ four of them supporting the superiority of OFD/PRF versus OFD, and one the superiority of BG/PRF versus BG.

The formation of a blood clot alone has been shown to be one of the key necessary features in order for periodontal regeneration to take place, as long as bacterial pathogens have been completely eliminated. Evidence from the literature suggests that blood clot formation alone is enough to treat a number of intrabony defects where space maintenance is not an issue (two‐ or three‐walled defects).^105^ PRF therefore acts in a similar fashion whereby the fibrin scaffold can be inserted into the periodontal pocket acting as a stable clot, with significant increases in platelets, leukocytes, and growth factors. While periodontal regeneration remains complex due to the number of tissues needed to be regenerated (new cementum, periodontal ligament, and alveolar bone), as well as the fact Sharpey's fibers need to be oriented functionally to support the tooth apparatus, it remains difficult to assess whether PRF actually leads to true periodontal regeneration since no human histological evidence exists to date on the topic (despite over 55 RCTs having been performed). Nevertheless, it is known that periodontal disease is caused by bacterial pathogens and an increase in regenerative growth factors and cells, as well as its incorporation of defense‐fighting leukocytes is certainly hypothesized to favor defect resolution and potentially mitigate tissue inflammation. Furthermore, angiogenesis is an important factor for tissue regeneration, and PRF releases a number of pro‐angiogenic and pro‐fibrotic agents capable of further speeding periodontal tissue repopulation.^105^, ^106^

The biological advantages of PRF have been shown to act locally by quickly stimulating a large number of cell types by influencing their recruitment, proliferation, and/or differentiation. These have previously been shown to include endothelial cells, gingival fibroblasts, chondrocytes, and osteoblasts, thereby having the potential effect to act locally and affect various cell types.^107^, ^108^ Thus, PRF may prove beneficial for the regeneration of specific tissues such as the periodontium since several cell types and tissue types are required to regenerate in order for periodontal regeneration to occur. While it is known that beneficial effects of PRF may partially be due to the large number of secreted autologous blood‐derived growth factors, it remains interesting to point out the fact that rhPDGF (which is approved by the FDA) has been one of the main recombinant growth factors sold to date in North America for the regeneration of periodontal tissues.^109^, ^110^, ^111^, ^112^ Although recombinant proteins have a regenerative potential well documented in the literature,^113^, ^114^, ^115^ their associated costs and other secondary adverse effects including biocompatibility, lower stability, and potential swelling, may favor the use of autologous PRF.^116^, ^117^ Future comparative studies including a cost–benefit analysis between both modalities remains necessary.

All therapeutic modalities with addition of PRF to their surgical approach (Groups I and IV) demonstrated better outcomes apart from the studies comparing OFD/EMD versus OFD/EMD/PRF. Treatment modalities comparing PRF alone to other regenerative strategies (Group III) found similar clinical outcomes between both groups. Each of the therapeutic modalities utilizing PRF with addition of small biomolecules (Group IV) found improved clinical outcomes with addition of either metformin, bisphosphonates, and statins. It thus remains interesting to note that generally speaking, the additional use of PRF tends to favor regenerative outcomes of IBDs, and addition of small biomolecules may further improve such outcomes. Future research investigating more precisely when PRF should be utilized in combination approaches versus as a sole regenerative modality needs further clarification.

Several research topics also remain at the forefront of needed study in this space. As previously mentioned, it remains interesting to point out that no single study has characterized PRF at the histological level in a well‐characterized human study. It has already been well established in the literature that PRF favors soft tissue wound healing when compared to hard tissues.^118^ Since periodontitis is not only characterized by PDL breakdown but also that of cementum and alveolar bone, the regenerative potential of each of these tissues needs to be further characterized via histological evaluation, ideally in human studies. Noteworthy as well, the type of the surgical technique (flap choice—minimally invasive surgical techniques), intrabony defect depth (two‐wall and three‐wall defects may respond differently to the treatment modality), tooth type, defect morphology, and clinician factors are all additional important factors that need further investigation for better addressing which combination therapy my best serve the treatment of each defect type. A recent publication titled “Do autologous platelet concentrates (APCs) have a role in intraoral bone regeneration? A critical review of clinical guidelines on decision‐making process” published in this same issue of *Periodontology 2000* highlights some of these findings.^119^

### 4.7 Better understanding of platelet concentrates

In a previous publication by our group titled: “Controversies related to scientific report describing g‐forces from studies on platelet‐rich fibrin: necessity for standardization of relative centrifugal force values,”^120^ the topic of properly reporting PRF protocols was discussed in great detail. The present list of studies found large variability to protocols utilized, many of which were utilized on different‐sized centrifuges. The same protocol utilized on a larger centrifuge will produce a larger RCF (g‐force) value and thus completely disruption the cell layer separation.^120^ For these reasons, in the year 2019 attempts have been made to raise awareness regarding the proper reporting of studies related to PRF.^121^ It remains of concern to report many studies that omit much important data during the preparation of PRF. Table 2 demonstrates that 13 different protocols were utilized in these RCTs, many protocols only ever utilized in one study with little scientific reasoning or validation for the choice of their centrifugation protocols. Noteworthy, 19 of 55 studies (35% of studies) failed to even report the company unit model, or size of their centrifuge and omitted any information at all regarding their centrifuge, largely impacting the reproducibility of their work.

**TABLE 2:** Platelet‐rich fibrin (PRF) protocols utilized throughout all studies.

Protocol utilized	Number of studies
3000 rpm × 10 min	32/55 studies
3000 rpm × 12 min	4/55 studies
2700 rpm × 12 min	4/55 studies
2800 rpm × 12 min	2/55 studies
1300 rpm × 8 min	2/55 studies
700 rpm × 3 min	2/55 studies
2500 rpm × 15 min	1/55 studies
2400 rpm × 2 min	1/55 studies
400 *g* × 10 min	2/55 studies
400 *g* × 12 min	1/55 studies
1000 *g* × 10 min	1/55 studies
373.3 *g* × 10 min	1/55 studies
NR	2/55 studies

Many improvements have been reported recently in terms of concentration in PRF formulations. It remains to be clinically investigated on the improvements of horizontal centrifugation versus fixed‐angle^99^, ^122^ and further optimization of PRF protocols. Furthermore, PRF tubes are critical for the success of PRF production,^123^ yet many studies have failed to report on their selected tube types utilized within their studies. A recent overview article titled: “Optimization of Platelet Rich Fibrin” aimed at critically assessing the production of PRF with more optimized/favorable methods.^124^

## 5 CONCLUSION

The use of PRF when compared to OFD alone led to statistically significant clinical improvements in PPD reduction, CAL gain, and RBF in intrabony defects when compared to OFD alone. The therapeutic modalities with addition of PRF (e.g. to a bone grafting material) demonstrated better outcomes apart from the studies comparing OFD/EMD versus OFD/EMD/PRF. Each of the treatment modalities comparing PRF alone to other regenerative strategies (Group III) found similar clinical outcomes between both groups. Each of the therapeutic modalities utilizing PRF with addition of small biomolecules primarily (Group V) found improved clinical outcomes with addition of either metformin, bisphosphonates, or statins. It thus remains interesting to note that generally speaking, the additional use of PRF tends to favor regenerative outcomes of IBDs, and addition of small biomolecules may further improve such outcomes. Limitations to this SR include the variability of the study groups included, limited number of RCTs per treatment group, and quality of the included studies based on the risk of bias analysis. Additional research investigating different protocols of PRF including human histological evaluation, as well as patient‐reported outcome measures (PROMs) remain highly necessary to further validate the PRF outcomes including various combination approaches.

## AUTHOR CONTRIBUTIONS

All authors made substantial contributions to the conception and design of the manuscript. RJM, MFK, and VM performed the literature search. PMJS and SJ performed the RoB analysis. RJM, MFK, VM, PMJS, and SJ contributed to the interpretation of the data. KJ provided clinical case documentation. All authors drafted the work and revised it critically for important intellectual content, agreed to be accountable for all aspects of the study design and its content, and approved the final submitted version.

## CONFLICT OF INTEREST STATEMENT

Richard J. Miron holds intellectual property on PRF (patents and trademarks) and is the founder of Miron Research and Development in Dentistry LLC involved with PRF.

## ETHICAL APPROVAL

No ethics approval was required for this study since it was a systematic review.

## INFORMED CONSENT

No informed consent was required.

## Supporting information


Tables S1–S6.

Appendix S1.

## Data Availability

Data sharing is not applicable to this article as no new data were created or analyzed in this study.
