# Supplementary material for: Autogenous platelet concentrates for treatment of intrabony defects—A systematic review with meta‐analysis
Source: Periodontol 2000. 2024 Oct 19;97(1):153–90. doi: 10.1111/prd.12598 (PMC11808470; doi:10.1111/prd.12598)
Supplement: Supplementary file 1 — Tables S1–S6. [file PRD-97-153-s001.docx]

**Supplementary Tables**

**SUPPL. TABLE 1:** RCTs comparing open flap debridement (OFD) with versus without the application of PRP / PRGF in the treatment of intra-bony defects (for some studies only 2 of the examined arms were considered). Only statistically significant differences are mentioned.

| **Article** | **Study**  **design** | **subjects:**  **gender/age**  **% smokers** | **defects: n**  **type**  **follow-up** | **centrifuge**  **RPM or g force**  **minutes** | **Groups** | **Impact on intra-bony parameters** |
| --- | --- | --- | --- | --- | --- | --- |
| **A: PRP** | | | | | | |
| **Using PRP gel only to “fill” the intra-bony defect** | | | | | | |
| Pradeep et al. 2012^1^ | RCT  parallel | 50 patients  mean age: 36.8  smokers = 0% | 90 defects  3 walls  9 months | -  3.000 rpm  10 min | C: OFD  T: OFD + PRP gel  *2 of 3 arms* | ***OFD + PRP gave:***  **> PPD reduction (3.8 *vs.* 3.0 mm)**  **> IBD depth reduction (2.7 *vs.* 0.1 mm)**  **> IBD fill (57 *vs.* 2%)** |
| Agarwal et al. 2016^2^ | RCT  split-mouth | 10 patients  ♀ = 3 / ♂ = 7  age: NR  smokers = 0% | 20 defects  3 walls  12 months | -  3.000 rpm  10 min | C: OFD  T: OFD + PRP gel  *2 of 3 arms* | ***OFD + PRP gave:***  **> PPD reduction (4.8 *vs.* 2.7mm)**  **> CAL gain (4.1 *vs.* 1.3 mm)**  **> Defect depth red. (2.1 *vs.* 0.3 mm)** |
| **B: PRGF** | | | | | | |
| **Using PRGF gel only to “fill” the intra-bony defect** | | | | | | |
| Khalifehzadeh et al. 2019^3^ | RCT  parallel | 8 patients  ♀ = 5 / ♂ = 3  mean age: 35 | 12 sites  2 walls  6 months | Endoret System IV  460 g  8 min | C: OFD  T: OFD + PRGF  *2 of 4 arms* | ***the addition of PRP gave:***  no significant difference |

**SUPPL. TABLE 2:** RCTs evaluating the beneficial impact of adding PRP to a “xenograft” as defect filler during OFD (for some studies only 2 of the examined arms were considered). Only statistically significant parameters are mentioned.

| **Article** | **Study**  **design** | **subjects:**  **gender/age**  **% smokers** | **defects: n**  **type**  **follow-up** | **centrifuge**  **RPM or g force**  **minutes** | **Groups** | **Impact on intra-bony parameters** |
| --- | --- | --- | --- | --- | --- | --- |
| **xenograft + PRP** | | | | | | |
| Hanna et al. 2004^4^ | RCT  split-mouth | 13 patients  ♀ = 5 / ♂ = 8  age: 37-74  smokers = 8% | 26 defects  2/3 wall  6 months | Smart PReP  -  - | C: OFD + xenograft  T: OFD + xenograft + PRP | ***the addition of PRP gave:***  **> PPD reduction (3.5 *vs.* 2.5 mm)**  **> CAL gain (3.2 *vs.* 2.3 mm)** |
| Ouyang et al. 2006^5^ | RCT  parallel | 10 patients  ♀ = 5 / ♂ = 5  age: 27-45  smokers = 0% | 17 defects  2/3 wall  12 months | Universal 16R  1220 rpm for 15 min  3600 rpm for 15 min | C: OFD + xenograft  T: OFD + xenograft + PRP | ***the addition of PRP gave:***  **> PPD reduction (4.8 *vs.* 3.5 mm)**  **> CAL gain (4.5 *vs.* 2.9 mm)**  **> bone fill (73 *vs.* 47 %)** |
| Döri et al. 2009^6^ | RCT  parallel | 30 patients  ♀ = 9 / ♂ = 21  age: 28-65  smokers = 0% | 30 defects  1/2 wall  12 months | -  2400 rpm for 10 min  3600 rpm for 15 min | C: OFD + xenograft  T: OFD + xenograft + PRP | ***the addition of PRP gave:***  no significant difference |
| **xenograft + PRGF** | | | | | | |
| no papers available | | | | | | |

**SUPPL. TABLE 3**: RCTs evaluating the beneficial impact of adding PRP to a “bone substitute” as defect filler during OFD (for some studies only 2 of the examined arms were considered). Only statistically significant parameters are mentioned.

| **Article** | **Study**  **design** | **subjects:**  **gender/age**  **% smokers** | **defects: n**  **type**  **follow-up** | **centrifuge**  **RPM or g force**  **minutes** | **Groups** | **Impact on intra-bony parameters** |
| --- | --- | --- | --- | --- | --- | --- |
| **Bone Substitute + PRP** | | | | | | |
| Okuda et al. 2005^7^ | RCT  parallel | 70 patients  ♀ = 49 / ♂ = 21  mean age: 55.5  smokers = 0% | 70 defects  1/2/3 wall  12 months | -  2400 rpm for 10 min  3600 rpm for 15 min | C: OFD + HA  T: OFD + HA + PRP | ***the addition of PRP gave:***  **> PPD reduction (4.7 *vs.* 3.7 mm)**  **> CAL gain (3.4 *vs.* 2.0 mm)** |
| Demir et al. 2007^8^ | RCT  parallel | 29 patients  ♀ = 16 / ♂ = 13  mean age: 36.0  smokers = 9 | 29 defects  1/2/3 wall  9 months | -  200 g for 10 min  250 g for 10 min | C: OFD + bioactive glass  T: OFD + bioactive glass + PRP | ***the addition of PRP gave:***  no significant difference |
| Piemontese et al. 2008^9^ | RCT  parallel | 60 patients  ♀ = 29 / ♂ = 31  age: 47 - 72  smokers = 0% | 60 defects  2/3 wall  12 months | SmartPReP  2400 rpm for 10 min  3600 rpm for 15 min | C: OFD + DFDBA  T: OFD + DFDBA + PRP | ***the addition of PRP gave:***  **> PPD reduction (4.3 *vs.* 2.6 mm)**  **> CAL gain (3.5 *vs.* 2.3 mm)** |
| Kaushick et al. 2011^10^ | RCT  split-mouth | 10 patients  gender: NR  age: 20 - 50  smokers = 0% | 20 defects  2/3 wall  6 months | -  5000 rpm for 10 min  2000 rpm for 10 min | C: OFD + HA/ß-TCP  T: OFD + HA/ßTCP + PRP | ***the addition of PRP gave:***  **> PPD reduction (4.3 *vs.* 3.3 mm)**  **> CAL gain (4.4 *vs.* 2.9 mm)** |
| Hassan et al. 2012^11^ | RCT  split-mouth | 12 patients  ♀ = 29 / ♂ = 31  mean age: 41.4  smokers = 0% | 24 defects  2 wall  12 months | -  200 g for 20 min  400 g for 10 min | C: OFD + AB  T: OFD + AB + PRP | ***the addition of PRP gave:***  **> PPD reduction (4.9 *vs.* 4.4 mm)**  **> CAL gain (3.8 *vs.* 2.9 mm)** |
| Ozdemir & Okte 2012^12^ | RCT  split-mouth | 14 patients  ♀ = 5 / ♂ = 9  mean age: 48.9  smokers = 0% | 28 defects  3 wall  6 months | Heraeus Labofuge  2400 rpm for 10 min  3600 rpm for 15 min | C: OFD + ß-TCP  T: OFD + ßTCP + PRP | ***the addition of PRP gave:***  no significant difference |
| Gupta 2014^13^ | RCT  split-mouth | 10 patients  gender: NR  age: NR  smokers = 0% | 20 defects  NR wall  12 months | -  1200 rpm for 20 min  2000 rpm for 15 min | C: OFD + HA  T: OFD + HA + PRP | ***the addition of PRP gave:***  **> PPD reduction (3.4 *vs.* 1.9 mm)**  **> CAL gain (3.0 *vs.* 1.3 mm)** |
| Agarwal & Gupta 2014^14^ | RCT  split-mouth | 24 patients  ♀ = 10 / ♂ = 14  age: 30 - 65  smokers = 0% | 48 defects  1/2 wall  12 months | -  2400 rpm for 10 min  3600 rpm for 15 min | C: OFD + DFDBA  T: OFD + DFDBA + PRP | ***the addition of PRP gave:***  **> CAL gain (3.1 *vs.* 2.4 mm)**  **> rBL gain (3.0 *vs.* 2.3 mm)** |
| Shukla et al. 2016^15^ | RCT  split-mouth | 20 patients  ♀ = 7 / ♂ = 13  mean age: 40  smokers = 0% | 40 defects  NR wall  9 months | -  2000 rpm for 15 min  3000 rpm for 15 min | C: OFD + CPS  T: OFD + CPS + PRP | ***the addition of PRP gave:***  no significant difference |
| Agarwal et al. 2016^2^ | RCT  split-mouth | 10 patients  ♀ = 3 / ♂ = 7  age: NR  smokers = 0% | 20 defects  3 wall  12 months | -  3000 rpm for 10 min | C: OFD + DFDBA  T: OFD + DFDBA + PRP  *2 of 3 arms* | ***the addition of PRP gave:***  **> PPD reduction (4.9 *vs.* 2.7 mm)**  **> CAL gain (4.2 *vs.* 1.3 mm)**  **> Defect depth red. (3.2 *vs.* 0.3 mm)** |
| Garg et al. 2017^16^ | RCT  parallel | 24 patients  ♀ = 15 / ♂ = 9  age: 28 - 47  smokers = 0% | 24 defects  3 wall  9 months | -  2400 rpm for 10 min  3600 rpm for 15 min | C: OFD + HA/ß-TCP  T: OFD + HA/ß-TCP + PRP | ***the addition of PRP gave:***  **> PPD reduction (4.1 *vs.* 3.3 mm)**  **> CAL gain (3.9 *vs.* 3.0 mm)**  **> Defect fill (3.6 *vs.* 2.7 mm)** |

**SUPPL. TABLE 4:** RCTs evaluating the beneficial impact of adding PRP to a bone substitute/xenograft as defect filler covered by a GTR membrane during OFD (for some studies only 2 of the examined arms were considered). Only statistically significant parameters are mentioned.

| **Article** | **Study**  **design** | **subjects:**  **gender/age**  **% smokers** | **defects: n**  **type**  **follow-up** | **centrifuge**  **RPM or g force**  **minutes** | **Groups** | **Impact on intra-bony parameters** |
| --- | --- | --- | --- | --- | --- | --- |
| **Bone Substitute/GTR + PRP** | | | | | | |
| Christgau et al. 2006^17^ | RCT  split-mouth | 25 patients  ♀ = 15 / ♂ = 10  age: 26 - 62  smokers = 5 | 50 defects  NR wall  12 months | Prepared in the Division of Transfusion Medicine at the University Hospital of Regensburg | C: OFD + ß-TCP/GTRres  T: OFD + ß-TCP/GTRres + PRP | ***the addition of PRP gave:***  no significant difference |
| Döri et al. 2007a^18^ | RCT  parallel | 30 patients  ♀ = 16 / ♂ = 14  age: 28 - 56  smokers = 0% | 30 defects  1/2/3 wall  12 months | Curasan PRP kit  2400 rpm for 10 min  3600 rpm for 15 min | C: OFD + NBM/GTRres  T: OFD + NBM/GTRres + PRP | ***the addition of PRP gave:***  no significant difference |
| Döri et al. 2007b^19^ | RCT  parallel | 24 patients  ♀ = 14 / ♂ = 10  age: 26 - 55  smokers = 0% | 24 defects  1/2 wall  12 months | Curasan PRP kit  2400 rpm for 10 min  3600 rpm for 15 min | C: OFD + ABBM/GTRnon-res  T: OFD + ABBM/GTRnon-res + PRP | ***the addition of PRP gave:***  no significant difference |
| Döri et al. 2008a^20^ | RCT  parallel | 28 patients  ♀ = 16 / ♂ = 12  age: 28 - 58  smokers = 0% | 28 defects  1/2/3 wall  12 months | Curasan PRP kit  2400 rpm for 10 min  3600 rpm for 15 min | C: OFD + ß-TCP/GTRnon-res  T: OFD + ß-TCP/GTRnon-res + PRP | ***the addition of PRP gave:***  no significant difference |
| Camargo et al. 2009^21^ | RCT  split-mouth | 23 patients  ♀ = 14 / ♂ = 9  age: 34 - 67  smokers = 11 | 46 defects  2/3 wall  6 months | Curasan method  5600 rpm for 6 min | C: OFD + BPBM/GTRres  T: OFD + BPBM/GTRres + PRP | ***the addition of PRP gave:***  no significant difference |

**SUPPL. TABLE 5:** RCTs evaluating the beneficial impact of adding PRP to a EMD + xenograft as defect filler during OFD. Only statistically significant differences are mentioned.

| **Article** | **Study**  **design** | **subjects:**  **gender/age**  **% smokers** | **defects: n**  **type**  **follow-up** | **centrifuge**  **RPM or g force**  **minutes** | **Groups** | **Impact on intra-bony parameters** |
| --- | --- | --- | --- | --- | --- | --- |
| **EMD + Xenograft + PRP** | | | | | | |
| **Using PRP gel only to “fill” the intra-bony defect** | | | | | | |
| Döri et al. 2008b^22^ | RCT  parallel | 26 patients  ♀ = 14 / ♂ = 12  age: 32 - 56  smokers = 0% | 26 defects  1/2 walls  12 months | Curasan PRP kit  547 g for 10 min  1231 g for 15 min | C: OFD + EMD + NBM  T: OFD + EMD + MBM + PRP | ***the addition of PRP gave:***  no significant difference |

**SUPPL. TABLE 6:** RCTs evaluating the beneficial impact of adding PRGF to GTR as defect filler during OFD. Only statistically significant differences are mentioned.

| **Article** | **Study**  **design** | **subjects:**  **gender/age**  **% smokers** | **defects: n**  **type**  **follow-up** | **centrifuge**  **RPM or g force**  **minutes** | **Groups** | **Impact on intra-bony parameters** |
| --- | --- | --- | --- | --- | --- | --- |
| **GTR + PRGF** | | | | | | |
| **Using PRGF gel to “fill” the intra-bony defect** | | | | | | |
| Ravi et al. 2017^23^ | RCT  Split-mouth | 14 patients  ♀ = 9 / ♂ = 5  mean age: 43.2  smokers = 0% | 38 defects  2/3 walls  6 months | Remi CM-8 centrif.  460 g for 8 min | C: OFD + GTR  T: OFD + GTR + PRGF | ***the addition of PRGF gave:***  no significant difference |

**REFERENCES**

1. Pradeep A, Rao NS, Agarwal E, Bajaj P, Kumari M, Naik SB. Comparative evaluation of autologous platelet‐rich fibrin and platelet‐rich plasma in the treatment of 3‐wall intrabony defects in chronic periodontitis: a randomized controlled clinical trial. Journal of periodontology 2012;83:1499-1507.

2. Agarwal P, Chatterjee A, Gokhale S, Singh HP, Kandwal A. Evaluation of platelet-rich plasma alone or in combination with demineralized freeze dried bone allograft in treatment of periodontal infrabony defects: A comparative clinical trial. Journal of Indian Society of Periodontology 2016;20:42-47.

3. Khalifehzadeh S, Haghanifar S, Jenabian N, Kazemi S, Hajiahmadi M. Clinical and radiographic evaluation of applying 1% metformin biofilm with plasma rich in growth factor (PRGF) for treatment of two-wall intrabony periodontal defects: A randomized clinical trial. Journal of Dental Research, Dental Clinics, Dental Prospects 2019;13:51.

4. Hanna R, Trejo PM, Weltman RL. Treatment of intrabony defects with bovine‐derived xenograft alone and in combination with platelet‐rich plasma: A randomized clinical trial. Journal of periodontology 2004;75:1668-1677.

5. Ouyang X-y, Qiao J. Effect of platelet-rich plasma in the treatment of periodontal intrabony defects in humans. Chinese medical journal 2006;119:1511-1521.

6. Döri F, Kovacs V, Arweiler NB, et al. Effect of platelet‐rich plasma on the healing of intrabony defects treated with an anorganic bovine bone mineral: A pilot study. Journal of periodontology 2009;80:1599-1605.

7. Okuda K, Tai H, Tanabe K, et al. Platelet‐rich plasma combined with a porous hydroxyapatite graft for the treatment of intrabony periodontal defects in humans: A comparative controlled clinical study. Journal of periodontology 2005;76:890-898.

8. Demir B, Şengün D, Berberoğlu A. Clinical evaluation of platelet‐rich plasma and bioactive glass in the treatment of intra‐bony defects. Journal of clinical periodontology 2007;34:709-715.

9. Piemontese M, Aspriello SD, Rubini C, Ferrante L, Procaccini M. Treatment of periodontal intrabony defects with demineralized freeze‐dried bone allograft in combination with platelet‐rich plasma: A comparative clinical trial. Journal of periodontology 2008;79:802-810.

10. Kaushick BT, Jayakumar N, Padmalatha O, Varghese S. Treatment of human periodontal infrabony defects with hydroxyapatite+ β tricalcium phosphate bone graft alone and in combination with platelet rich plasma: a randomized clinical trial. Indian Journal of Dental Research 2011;22:505-510.

11. Hassan K, Alagl A, Abdel-Hady A. Torus mandibularis bone chips combined with platelet rich plasma gel for treatment of intrabony osseous defects: clinical and radiographic evaluation. International journal of oral and maxillofacial surgery 2012;41:1519-1526.

12. Özdemir B, Ökte E. Treatment of intrabony defects with beta‐tricalciumphosphate alone and in combination with platelet‐rich plasma. Journal of Biomedical Materials Research Part B: Applied Biomaterials 2012;100:976-983.

13. Gupta G. Clinical and radiographic evaluation of intra-bony defects in localized aggressive periodontitis patients with platelet rich plasma/hydroxyapatite graft: A comparative controlled clinical trial. Contemporary clinical dentistry 2014;5:445-451.

14. Agarwal A, Gupta ND. Platelet-rich plasma combined with decalcified freeze-dried bone allograft for the treatment of noncontained human intrabony periodontal defects: a randomized controlled split-mouth study. International Journal of Periodontics & Restorative Dentistry 2014;34.

15. Shukla S, Chug A, Mahesh L, Grover HS. Effect of Addition of Platelet-rich Plasma to Calcium Phosphosilicate Putty on Healing at 9 Months in Periodontal Intrabony Defects. The journal of contemporary dental practice 2016;17:230-234.

16. Garg K, Srivastava R, Verma PK, Gautam A, Tripathi V, Agarwal S. Clinical evaluation of platelet rich plasma when combined with an alloplastic bone graft material in the treatment of intrabony periodontal defects. Saudi Journal of Oral Sciences 2017;4:33-40.

17. Christgau M, Moder D, Wagner J, et al. Influence of autologous platelet concentrate on healing in intra‐bony defects following guided tissue regeneration therapy: A randomized prospective clinical split‐mouth study. Journal of clinical periodontology 2006;33:908-921.

18. Döri F, Huszár T, Nikolidakis D, Arweiler NB, Gera I, Sculean A. Effect of platelet‐rich plasma on the healing of intra‐bony defects treated with a natural bone mineral and a collagen membrane. Journal of clinical periodontology 2007;34:254-261.

19. Döri F, Huszár T, Nikolidakis D, Arweiler NB, Gera I, Sculean A. Effect of platelet‐rich plasma on the healing of intrabony defects treated with an anorganic bovine bone mineral and expanded polytetrafluoroethylene membranes. Journal of periodontology 2007;78:983-990.

20. Döri F, Huszar T, Nikolidakis D, et al. Effect of platelet‐rich plasma on the healing of intrabony defects treated with beta tricalcium phosphate and expanded polytetrafluoroethylene membranes. Journal of Periodontology 2008;79:660-669.

21. Camargo PM, Lekovic V, Weinlaender M, Divnic‐Resnik T, Pavlovic M, Kenney EB. A surgical reentry study on the influence of platelet‐rich plasma in enhancing the regenerative effects of bovine porous bone mineral and guided tissue regeneration in the treatment of intrabony defects in humans. Journal of periodontology 2009;80:915-923.

22. Döri F, Nikolidakis D, Huszar T, Arweiler NB, Gera I, Sculean A. Effect of platelet‐rich plasma on the healing of intrabony defects treated with an enamel matrix protein derivative and a natural bone mineral. Journal of Clinical Periodontology 2008;35:44-50.

23. Ravi S, Malaiappan S, Varghese S, Jayakumar ND, Prakasam G. Additive effect of plasma rich in growth factors with guided tissue regeneration in treatment of intrabony defects in patients with chronic periodontitis: a split‐mouth randomized controlled clinical trial. Journal of periodontology 2017;88:839-845.
