# Supplementary material for: Autogenous platelet concentrates for treatment of intrabony defects—A systematic review with meta‐analysis
Source: Periodontol 2000. 2024 Oct 19;97(1):153–90. doi: 10.1111/prd.12598 (PMC11808470; doi:10.1111/prd.12598)

Unique ID	Study ID	Experimental	Comparator	Outcome	Weight	D1	D2	D3	D4	D5	Overall
Abdulrahman 2022	Vertical	NA	NA	NA	1	+	+	+	+	+	+
Aggour 2017	Vertical	NA	NA	NA	1	!	!	+	+	+	!
Agarwal 2016	Vertical	NA	NA	NA	1	!	+	+	+	+	!
Agarwal 2017	Vertical	NA	NA	NA	1	!	+	+	!	+	!
Ajwani 2015	Vertical	NA	NA	NA	1	!	+	+	+	+	!
Alshoiby 2023	Vertical	NA	NA	NA	1	+	+	+	+	+	+
Atchuta 2020	Vertical	NA	NA	NA	1	!	+	+	!	+	!
Aydemir-Turkal 2016	Vertical	NA	NA	NA	1	+	+	+	+	+	+
Baghele 2023	Vertical	NA	NA	NA	1	+	+	+	+	+	+
Bahammam 2021	Vertical	NA	NA	NA	1	+	+	+	+	+	+
Bajaj 2017	Vertical	NA	NA	NA	1	!	+	+	+	+	!
Bansal 2013	Vertical	NA	NA	NA	1	!	+	+	!	+	!
Bodhare 2019	Vertical	NA	NA	NA	1	!	+	+	+	+	!
Chadwick 2016	Vertical	NA	NA	NA	1	+	+	+	+	+	+
Chandradas 2016	Vertical	NA	NA	NA	1	+	+	+	+	+	+
Chatterjee 2017	Vertical	NA	NA	NA	1	!	+	+	!	+	!
Chaudhary 2023	Vertical	NA	NA	NA	1	+	+	+	!	+	!
Csifó-Nagy 2021	Vertical	NA	NA	NA	1	!	+	+	+	+	!
Elbehwashy 2021	Vertical	NA	NA	NA	1	+	+	+	+	+	+
Elgendy 2015	Vertical	NA	NA	NA	1	!	!	+	!	+	!
Galav 2016	Vertical	NA	NA	NA	1	!	+	+	!	+	!
Gamal 2016	Vertical	NA	NA	NA	1	!	+	+	+	+	!
Gautam 2022	Vertical	NA	NA	NA	1	!	+	+	!	+	!
Goyal 2020	Vertical	NA	NA	NA	1	+	+	+	+	+	+
Gummaluri 2020	Vertical	NA	NA	NA	1	!	+	+	+	+	!
Gupta 2014	Vertical	NA	NA	NA	1	!	!	+	!	+	!
Hazari 2021	Vertical	NA	NA	NA	1	!	+	+	!	+	!
Joseph 2012	Vertical	NA	NA	NA	1	!	+	+	+	+	!
Kanoriya 2016	Vertical	NA	NA	NA	1	!	+	+	+	+	!
Lekovic 2012	Vertical	NA	NA	NA	1	-	+	+	+	+	-
Liu 2021	Vertical	NA	NA	NA	1	!	+	+	+	+	!
Martande 2016	Vertical	NA	NA	NA	1	!	+	+	+	+	!
Mathur 2015	Vertical	NA	NA	NA	1	!	+	+	-	!	-
Mubarak 2023	Vertical	NA	NA	NA	1	+	+	+	+	+	+
Naqvi 2017	Vertical	NA	NA	NA	1	!	+	+	+	+	!
Panda 2016	vertical	NA	NA	NA	1	+	+	+	+	+	+
Paolantonio 2020	Vertical	NA	NA	NA	1	+	+	+	+	+	+
Patel 2017	Vertical	NA	NA	NA	1	!	+	+	+	+	!
Pavani 2021	Vertical	NA	NA	NA	1	!	!	+	+	+	!
Pham 2021	Vertical	NA	NA	NA	1	!	+	+	!	+	!
Pradeep 2012	Vertical	NA	NA	NA	1	!	+	+	+	+	!
Pradeep 2015	Vertical	NA	NA	NA	1	!	+	+	+	+	!
Pradeep 2016	Vertical	NA	NA	NA	1	!	+	+	+	+	!
Pradeep 2017	Vertical	NA	NA	NA	1	!	+	+	+	+	!
Rexhepi 2021	Vertical	NA	NA	NA	1	+	+	+	+	+	+
Saravanan 2019	Vertical	NA	NA	NA	1	!	!	+	-	+	-
Sezgin 2017	Vertical	NA	NA	NA	1	!	+	+	+	+	!
Sharma 2011	Vertical	NA	NA	NA	1	!	+	+	+	+	!
Thalaimalai 2020	Vertical	NA	NA	NA	1	!	+	+	+	+	!
Thetay 2021	Vertical	NA	NA	NA	1	!	+	+	+	+	!
Thorat 2011	Vertical	NA	NA	NA	1	!	+	+	+	+	!
Thorat 2017	Vertical	NA	NA	NA	1	!	+	+	+	+	!
Ugale 2023	Vertical	NA	NA	NA	1	+	+	+	+	+	+
Ustaoğlu 2020	Vertical	NA	NA	NA	1	!	+	+	+	+	!
Yajamanya 2017	Vertical	NA	NA	NA	1	!	+	+	-	+	-

+	Low risk
!	Some concerns
-	High risk
D1	Randomisation process
D2	Deviations from the intended interventions
D3	Missing outcome data
D4	Measurement of the outcome
D5	Selection of the reported result

	Randomization process	Deviations from intended interventions	Mising outcome data	Measurement of the outcome	Selection of the reported result	Overall Bias
Total number of study = 55						
Low risk	27.3	90.9	100	74.5	98.2	25.5
Some concerns	70.9	9.1	0	20.0	1.8	67.3
High risk	1.8	0	0	5.5	0	7.3

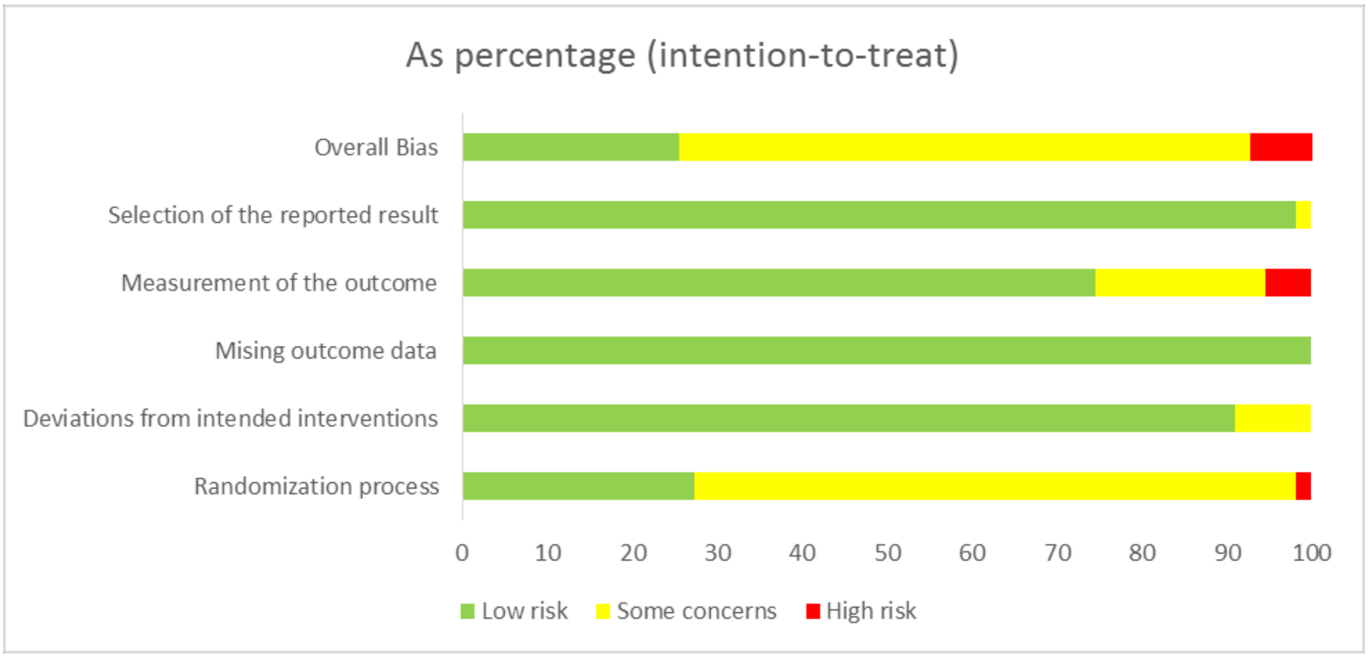

Supplement: Supplementary file 2 — Appendix S1. [file PRD-97-153-s002.pdf]
